# Brazilian Science between National and Foreign Journals: Methodology for Analyzing the Production and Impact in Emerging Scientific Communities

**DOI:** 10.1371/journal.pone.0155148

**Published:** 2016-05-12

**Authors:** Letícia Strehl, Luciana Calabró, Diogo Onofre Souza, Lívio Amaral

**Affiliations:** 1 Biblioteca Central, Universidade Federal do Rio Grande do Sul, Porto Alegre, Rio Grande do Sul, Brazil; 2 Programa de Pós-Graduação em Educação em Ciências: Química da Vida e Saúde. Universidade Federal do Rio Grande do Sul, Porto Alegre, Rio Grande do Sul, Brazil; 3 Departamento de Bioquímica, Instituto de Ciências Básicas da Saúde, Universidade Federal do Rio Grande do Sul, Porto Alegre, Rio Grande do Sul, Brazil; 4 Departamento de Física, Instituto de Física, Universidade Federal do Rio Grande do Sul, Porto Alegre, Rio Grande do Sul, Brazil; Max Planck Society, GERMANY

## Abstract

In recent decades, we have observed an intensification of science, technology and innovation activities in Brazil. The increase in production of scientific papers indexed in international databases, however, has not been accompanied by an equivalent increase in the impact of publications. This paper presents a methodology for analyzing production and the impact of certain research areas in Brazil related to two aspects: the origin of the journals (national or foreign) and international collaboration. These two variables were selected for being of particular importance in understanding the context of scientific production and communication in countries with emerging economies. The sample consisted of papers written by Brazilian researchers in 19 subfields of knowledge published from 2002 to 2011, totaling 85,082 papers. To calculate the impact, we adopted a normalized indicator called the relative subfield citedness (Rw) using a window of 5 years to obtain measurements evaluated in 2 different years: 2007 and 2012. The data on papers and citations were collected from the Web of Science database. From the results, we note that most of the subfields have presented, from one quinquennium to another, improved performance in the world production rankings. Regarding publication in national and foreign journals, we observed a trend in the distribution maintenance of production of the subfields based on the origin of the journal. Specifically, for impact, we identified a lower Rw pattern for Brazilian papers when they were published in national journals in all subfields. When Brazilian products are published in foreign journals, we observed a higher impact for those papers, even surpassing the average global impact in some subfields. For international collaboration, we analyzed the percentage of participation of foreign researchers and the connection between collaboration and the impact of papers, especially emphasizing the distinction of hyperauthorship papers in terms of production and impact.

## Introduction

In recent decades, we have observed in countries with emerging economies an expansion of production activities at different levels, including the sectors of science, technology and innovation (ST & I) [1]. In Brazil, an important factor of expansion was the growth of the education system at the graduate level; this was the result of a national policy of both human resources formation and consolidation of the country’s ST & I infrastructure[2]. In 2002, 6,894 PhD degrees were awarded [3] and 16,240 papers were published in journals that were indexed in international databases[4]. Ten years later, these numbers were 13,912 and 44,001, respectively. Overall, among 239 countries, Brazil ranked 17th [4,5] in the world ranking of scientific paper production in 2002, ascending to 13th [5], or 14th [4] in 2011, depending on the database used.

However, in contrast to the improvement in Brazil’s scientific performance measured according to these parameters, it was possible to verify the low impact of the publications considering the number of citations by the scientific community. According to the Ranking of Countries produced by SCImago [5], Brazil is among the 30 most productive countries in science, at the 24th position in the ranking of citations per paper.

Among the possible hypothesis that have been developed to explain this phenomenon are: the difficulties of scientific excellence in a emerging and expanding system, the low international collaboration index of Brazilian researchers, the more regional nature of the research problems in certain knowledge areas that are mainly published in Brazilian journals. Regarding this last point, it is estimated that at least half of the papers published by Brazilian scientists are in journals that are published in Brazil [6].

It is worth mentioning that, likewise the growth of production, these issues about the low impact of the publications and the use of journals published in one’s own country are common advents in almost all emerging countries. As a consequence, the publication of journals in these countries plays a distinct role than in the leading countries in science, in which we can see, in addition, a commercial logic [7]. In opposition to this appeal, editing journals in the least developed countries meet the particular needs of improving scientific expertise in two distinct stages: first, for researchers, regarding the ability to communicate research results and second, for editors, in defining editorial policies for selecting important scientific results in specific areas of knowledge. This assumption implies that journals edited by emerging countries exert a bigger role for the development of the scientific community in their own country than in the international scientific community. This results in a clear degree of endogamy, as these journals publish more papers with authorships from their own country, characterizing them as national journals.

In recent years, the international databases began to contemplate a larger number of scientific journals published in less developed countries. Before this, the coverage of the work produced by these countries’ scientists was restricted almost exclusively to what was published in the developed countries. Thus, the indexation integrated the data on papers published in national journals with the data on publications considered to be in the mainstream of science, enabling the expansion of knowledge about the characteristics of the scientific production in emerging countries. According to Meneghini, “the establishment of national journals has created two parallel communication streams for scientists in emerging countries: publication in international journals—the selective route—and publication in national journals—the regional route” [7].

In this context, considering that the improvement in the international ranking of scientific production by Brazilian researchers was accompanied by an increase in the number of Brazilian scientific journals, the aim of this study was to ascertain the scientific production and the impact of some research areas in Brazil, considering the following:

1. Publication in national and foreign journals (defined as journals whose head offices are in Brazil and journals whose head offices are out of Brazil, respectively);
2. International collaboration (based on papers with Brazilian and foreign authorships).

Thus, with this objective, we expect to contribute to better understand the role of the national journals in Brazil’s position in the ranking in the international scenario over the last years. This also could contribute for a diffusion of a methodology potentially useful to other emerging countries to also understand the role of their national journals on their scientific development.

## Materials and Methods

### Samples

The subfields included in this study were those with at least 1 Brazilian journal indexed in the 2002 edition of the Journal Citation Report (JCR). With the application of this criterion, 19 subfields (Web of Science Subject Categories) were identified for the study, as follows: “Agriculture, Dairy & Animal Science”, “Agriculture, Multidisciplinary”, “Biochemistry & Molecular Biology”, “Biology”, “Chemistry, Multidisciplinary”, “Engineering, Chemical”, “Genetics & Heredity”, “Mathematics”, “Medicine, Research & Experimental”, “Microbiology”; “Neurosciences”, “Parasitology”, “Physics, Multidisciplinary”, “Psychiatry”, “Public Environmental & Occupational Health”, “Social Sciences, Interdisciplinary”, “Soil Science”, “Tropical Medicine” and “Veterinary Sciences”. Papers on the “Multidisciplinary Sciences” subfield met the adopted criterion but were not included in the sample because they did not identify and clearly define a thematic specialty. So, considering that the universe of analysis was restricted to papers published in journals of the 19 WoS Subject Categories, we constituted two samples as follows:

1. Sample 1 (Table 1). This sample included 252,270 papers (articles, proceedings, and reviews) published in 2012 and indexed by Web of Science (Wos). These papers were published in journals edited by 2 groups of 3 countries each. The first group, composed by the United States, England and Netherland, is responsible for editing the largest number of scientific journals in the world; and the second group, composed by China, Brazil and Russia, is responsible for editing the largest number of scientific journals among the emerging countries. In this sample, the endogamy level was evaluated, corresponding: country of publication / country of affiliation of the authors.
2. Sample 2. This sample included 85,082 papers (articles, proceedings, and reviews) published between 2002 and 2011 and indexed by WoS. These papers were published by authors with Brazilian affiliation. In this sample, the production, impact and international collaboration of Brazilian scientific papers published in national and foreign journals were characterized.

**Table 1:** Comparison of the endogamy level of journals published in different countries.

Main countries responsible for publishing journals	Number of papers (2012)	Endogamy level^a^
*International scope*		
**United States**	119,828	44.0%
**England**	75,293	10.9%
**Netherlands**	34,901	3.5%
*Emerging countries scope*		
**China**	10,879	87.5%
**Brazil**	5,978	80.4%
**Russia**	5,391	84.2%

^**a**^ % papers written by authors affiliated in the journals’ country of publication.

[Data sourced from Thomson Reuters Web of Science]

The Sample 1 data were collected from WoS on 12-23-2015 and the Sample 2 data were collected from WoS on 01-13-2014. We described in the S1 Appendix and S2 Appendix the procedures for obtaining the Sample 1 and Sample 2, respectively. However, considering the weekly update of the accessible data via WoS, the obtained figures will constantly depend on the access time to the database. The country of publication of the journals was defined according to the Source Publication Lists for Web of Science published by Thomson Reuters [8,9].

### Formula for calculating the impact: the Relative Subfield Citedness (Rw)

The comparative analysis of production and impact indicators of scientific activity has at least two challenges: a) different research areas have different production and citation patterns; and b) bibliographic databases have coverage variations over time, which can lead to a mistaken interpretation of changes in scientific performance, i.e., increasing production and impact indicators may be exclusively attributable to the increase in the number of journals indexed by the base.

For decades, this known issue has motivated scientometricians to search for formulas that produce relative impact indicators (normalized by a variety of parameters) that allow for absolute performance comparisons of communities of different areas at different periods of time without reservations. Vinkler [10] revises these indicators in an paper entitled in a funny way: "The case of scientometricians with the 'absolute relative' impact indicator".

Regardless of the approach, the starting point of all formulas is the average of citations by papers published by the researchers who constitute the focus of analysis. The controversy regards how to obtain the parameter that will serve for comparison and normalization and how to aggregate the publications by fields [10–13]. Among the numerous existing formulas [10], we use the indicator known in the literature as Relative Subfield Citedness (Rw). This normalization proposal was made decades ago by Vinkler [14] and has been used in different contexts.

According to Vinkler [15], “the Rw indicator relates the sum of citations obtained by a set of paper to a standard, which is independent of the discretion of the respective authors. The calculation of the standard requires the selection of a respective set of journals common in topic with the activity of the authors (papers) investigated”. Here, the normalization standard used is obtained by the average Garfield (impact) Factor (GF) of the journals in a specific subfield (WoS Subject Categories) and is obtained by applying the following formula:

$$\text{Rw = }\frac{{\sum^{\;}}_{\text{i = }1}^{\text{P}}{\text{c}}_{\text{i}}}{\begin{array}{cc} \text{GFm} & \cdot \text{ P} \end{array}}$$ (1)

where P is the total number of papers evaluated, c_i_ is the number of citations to the i-th paper evaluated, GFm is the mean Garfield factor of journals in the respective subfields according to a Journal Citation Report (JCR) specific edition.

To avoid misleading conceptions: the Impact Factor of the journals (GF) indicate the impact of the journals; the Number of Citations (c) received by the Papers (p) indicates the absolute impact of the papers. Based on these indexes, the Number of Citations (c) received by the Papers (p) normalized by Mean Impact Factor of Journals of the respective subfield (GFm) indicates relative impact of the papers (Rw). This index (Rw) cannot be confused with the use of GF as an indicator of the impact of the papers, an evaluation practice strongly questioned [16].

As a result, the Rw has the number 1 as a reference value for interpreting the impact of the different subfields. If Rw = 1, it can be concluded that the citedness of the papers in question is approximately the same as that of the subfield; If Rw< 1, the papers in question receive less appreciation from the scientific community than the average paper in that subfield; and if Rw> 1, the international scientific impact of the investigated publications is greater than that for the average paper in the subfield [14].

The advantage of the normalization method applied with Rw in comparison to other relative indicators derives from the fact that the factor GFm in the index Rw is calculated by averaging the GF of all journals classified in a specific WoS Subject Category. Other indicators only use the GF of the journals used for publication by the authors being assessed, limiting the comparison into a context defined by the authors themselves [10]. Thus, according to Vinkler [17], “the Rw represents the international impact of the papers more correctly, as it applies an external (international and objective) standard”. Furthermore, the GFm based in all journals of a specific WoS Subject Category is easier to be obtained, being calculated through a standard access to WoS and JCR.

However, two limitations should be highlighted in terms of choosing the Rw indicator as a tool for measuring the scientific paper’s impact. The first limitation refers to how to define the reference standard of the papers for standardizing the citations: we recognize that the field normalization based on WoS Subject Categories is criticized for being considered a very general classification. An extreme example of this problem is the papers published in journals of high visibility and generally classified as "Multidisciplinary Sciences", independently of the specificity of the paper. In order to attenuate this limitation, we removed from the sample papers published in journals of the subfield "Multidisciplinary Sciences", thus restricting the analysis to papers published in journals of specific subfields, which are more accurately contextualized by themes [11].

The second limitation refers to the fact that Rw is an indicator based on the mean of citations and not on the characteristics of the distribution of citations among publications. More specifically, the impact values measured by means may present distortions due to some highly cited scientific papers. However, a measurement that considers the characteristics of the distribution of citations create some challenges for an adequate definition of reference standard used for standardizing the impact data [18]. These challenges cannot be overcome in a study that utilize a standard access of the WoS for collecting data of scientific production classified in a so wide diversity of knowledge areas. Based on this brief discussion above, in our opinion, the use of the Rw as impact indicator allowed us to perform this study following our predefined study design.

To measure the impact, we used a 5-year citation window, adopting the same formula for the 2007 and 2012 editions of the JCR [19], that is, the ratio between the number of citations in 2007 (or 2012) to the published items in the last five years and the number of papers (source items) published in the same five years. We consider this period more favorable than the two years that are traditionally used given that on average, the half-life of journal citations in subfields is 7.57 years [19]. Production data and citations were obtained through “Analyze Results” and WoS “Citation Reports” [20].

Results for the production and impact of Brazilian papers are presented considering the aspects as previously mentioned: publication in national and foreign journals and international collaboration. Specifically, we emphasize that here we use scatter plots to facilitate descriptive analysis separate from their original purpose of representing correlations between variables.

## Results

Despite the international nature of science, Table 1 shows that there is a strong degree of endogamy in journals that are edited in emerging countries, which means the country in which the journals are published very often correspond to the country of affiliation of their authors.

Even in the US, the world leader in scientific production, we observed a relative low level of endogamy, well below the approximately 80% of endogamy observed in journals published by the emerging countries. The peculiarity of journals published by emerging countries suggests that the scientific production of these countries might have different characteristics depending on the place of publication. The following data on production, impact and international collaboration of the scientific production takes into account the possible need for this distinction. Thus, further analyzes aim the characterization of the Brazilian production when published in its own journals (national journals), and when published in journals edited by other countries (foreign journals).

**Fig 1:**
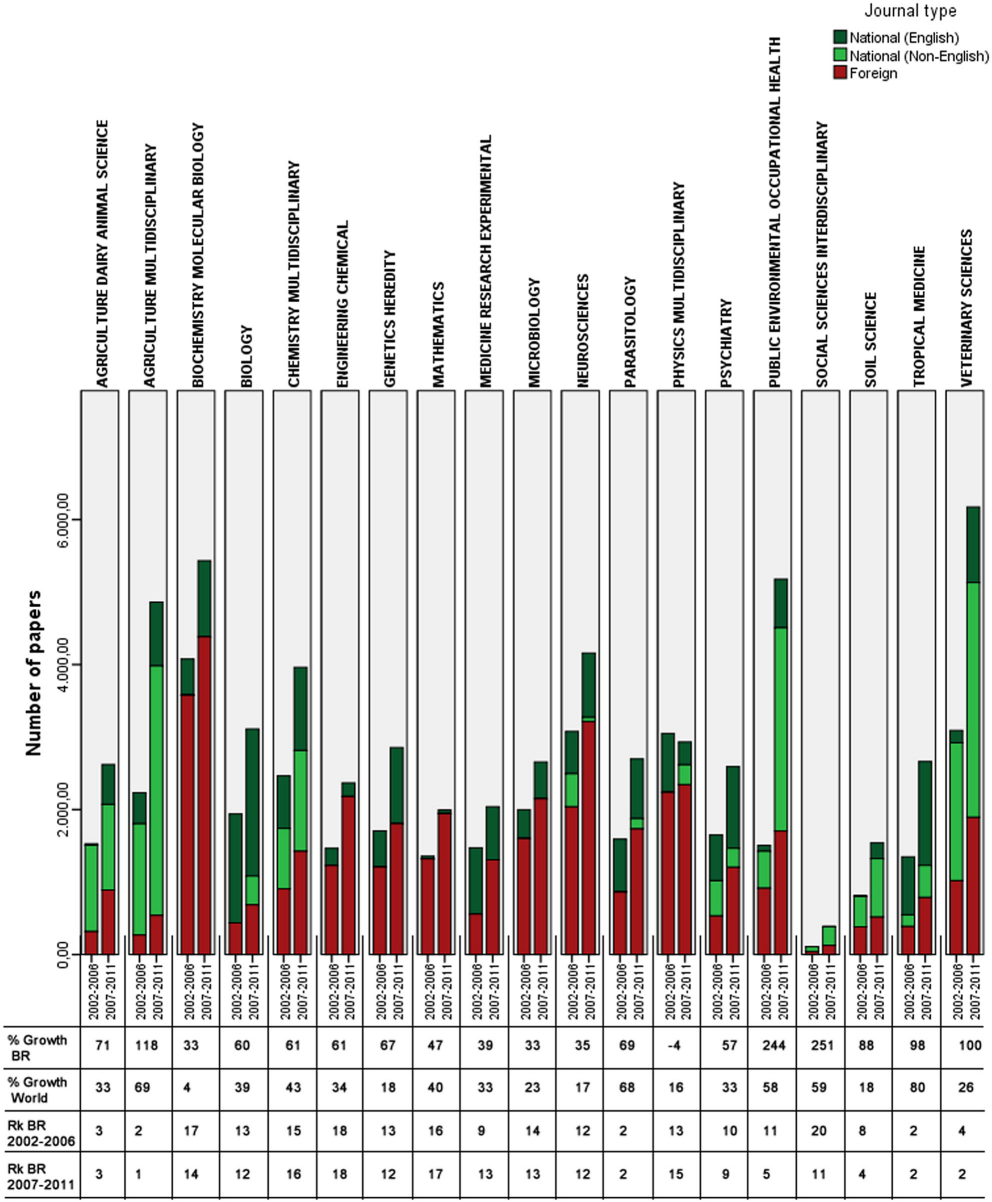
Brazilian scientific production by subfield, period and type of journal. % Growth BR, Percentage of growth in Brazilian scientific production indexed in WoS comparing two periods (2002–2006 and 2007–2011), % Growth World, Percentage of growth of the global scientific production indexed in WoS comparing two periods (2002–2006 and 2007–2011), Rk BR 2002–2006 Brazil’s world ranking in scientific production, 2002–2006, Rk BR 2007–2011 Brazil’s world ranking in scientific production, 2007–2011. [Data sourced from Thomson Reuters Web of Science].

In Fig 1, we observe that the percentage of growth in Brazil’s scientific production (% Growth BR) and the relative improvement in the country’s global rankings (Rk BR) varied depending on the subfield. In terms of absolute number of published papers, with the exception of the “Physics, Multidisciplinary” subfield, all subfields presented an increase in the number of published papers. The intensity of growth of each subfield is more properly measured when normalized by the increase in world production and the coverage of the WoS itself, as mentioned earlier. From this relativization, we analyzed the evolution of Brazil's position in the ranking of production by subfield (comparison Rk BR 2002–2006 and Rk BR 2007–2011).

In the first period (2002–2006), Brazil already had outstanding performance in the world ranking in 5 of 19 subfields: “Agriculture, Dairy & Animal Science”, “Agriculture, Multidisciplinary”, “Parasitology”, “Tropical Medicine” and “Veterinary Sciences”. In addition to these subfields, in the second period (Rk BR 2007–2011), Brazil also began to appear among the top five positions in the rankings on “Public Environmental & Occupational Health” and “Soil Science”. Although Brazil did not occupy the top of the ranking, “Social Sciences, Interdisciplinary” is another subfield with improved performance, moving 9 positions in the ranking from the first to the second period.

In addition to the position in the world ranking of scientific production, some of these subfields also showed growth rate clearly above the growth of global scientific production. This occurred in “Agriculture, Multidisciplinary”, “Parasitology”, “Public Environmental & Occupational Health”, “Social Sciences, Interdisciplinary” and “Tropical Medicine”.

The subfields in which Brazil presented a decrease in performance (Rk BR) during the period were: “Chemistry, Multidisciplinary”, “Mathematics”, “Medicine, Research & Experimental” and “Physics, Multidisciplinary”.

Regarding publication in national and foreign journals, we observed continued production distribution trends in the following subfields by type of journal (Brazilian or foreign). “Biochemistry & Molecular Biology”, “Engineering, Chemical”, “Mathematics”, “Microbiology” and “Physics, Multidisciplinary” are the subfields that proportionally publish most often in foreign journals, and, also proportionally, “Agriculture, Multidisciplinary” and “Biology” differ by predominantly publishing in national journals. More specifically, regarding production in national journals, another aspect that we highlight is the publication of papers in languages other than English. “Agriculture, Dairy & Animal Science”, “Agriculture, Multidisciplinary”, “Chemistry, Multidisciplinary”, “Public Environmental & Occupational Health”, “Social Sciences, Interdisciplinary” and “Veterinary Sciences” publish a significant number of papers in other languages, especially in Portuguese, reducing the international audience for the publications.

The only subfields that changed their profile publication by type of journal were “Medicine, Research & Experimental” and “Public Environmental & Occupational Health”. The first moved from predominantly publishing in national journals to foreign journals, and the other followed the opposite path.

**Fig 2:**
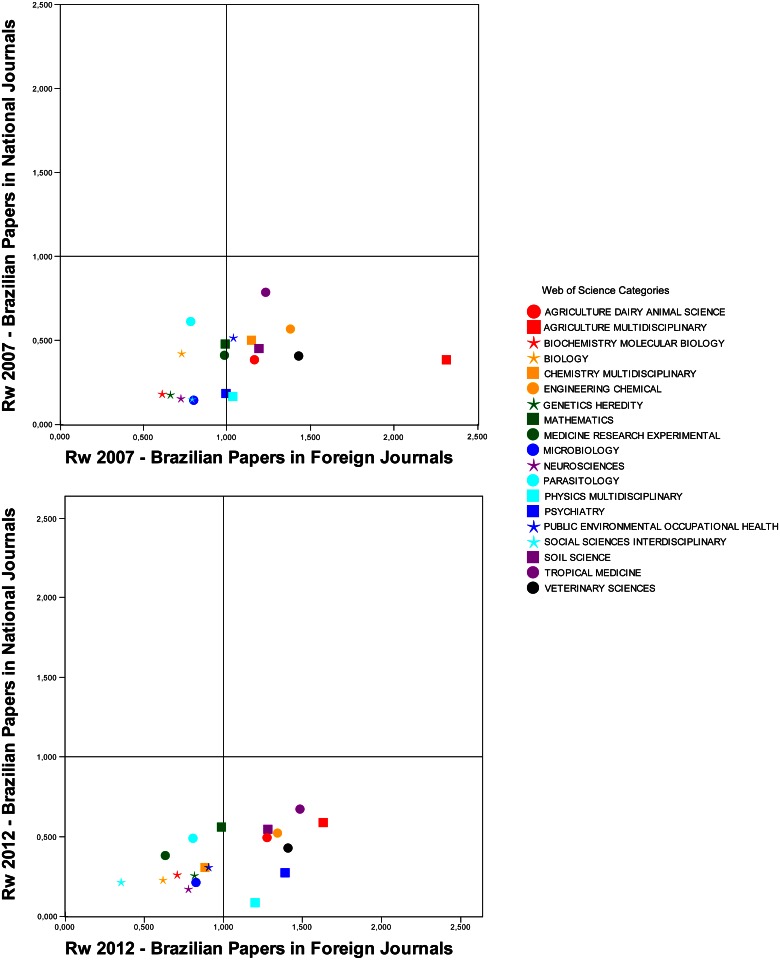
Brazilian Rw by type of journal in 2007 and 2012. [Data sourced from Thomson Reuters Web of Science].

Fig 2 shows the Rw values obtained for the different subfields by type of journal (national and foreign) at two different years (2007 and 2012). The intersection of both axes indicates the value Rw = 1, facilitating the comparison with the worldwide average impact of the respective subfield.

We observed that Brazilian scientific publication in national journals had less than average impact worldwide in all subfields in the 2 periods (Rw< 1). In a few subfields, production was greater than half the worldwide average (0.5 <R w< 1); this occurs in only two subfields in both periods: “Engineering, Chemical” and “Tropical Medicine” (the subfield responsible for the papers with the highest impact in national journals). “Agriculture, Multidisciplinary”, “Chemistry, Multidisciplinary”, “Mathematics”, “Parasitology”, “Public Environmental & Occupational Health” and “Soil Science” presented Rw measures within this standard in only one of the two years studied. Although there is a trend toward identifying the use of languages other than English as the cause of the low impact of national journals, our data show that this factor is not sufficient to explain the full problem. Even the national journals classified in subfields where the publications are mainly in English receive a low number of citations.

As expected, when Brazilian production is published in foreign journals, we observe a significant increase in the impact of papers in all subfields, many even surpassing the average worldwide impact (Rw> 1). Contrary to common sense, this superior impact to the worldwide average was identified mainly in subfields that publish predominantly in national journals; that is, when studies in these subfields are published in foreign journals, they have greater impact than the worldwide average. This occurred in both periods in the following subfields: “Agriculture, Dairy & Animal Science”, “Agriculture, Multidisciplinary”, “Soil Science”, “Tropical Medicine” and “Veterinary Sciences”. “Engineering, Chemical” and “Physics, Multidisciplinary” are the only two subfields that predominantly publish in foreign journals and have a production impact higher than the worldwide average when they publish in foreign journals. Three subfields presented Rw> 1 in only one of the two years: “Chemistry, Multidisciplinary” and “Public Environmental & Occupational Health” in 2007 and “Psychiatry” in 2012.

The comparison of both periods reveals a small oscillation in the impact of Brazilian scientific production in general. The most significant variations occurred in work published in foreign journals, especially in “Agriculture, Multidisciplinary” and “Social Sciences, Interdisciplinary”. Even with the decline in 2012, production in “Agriculture, Multidisciplinary” had higher than average impact in foreign journals in both periods studied.

**Fig 3:**
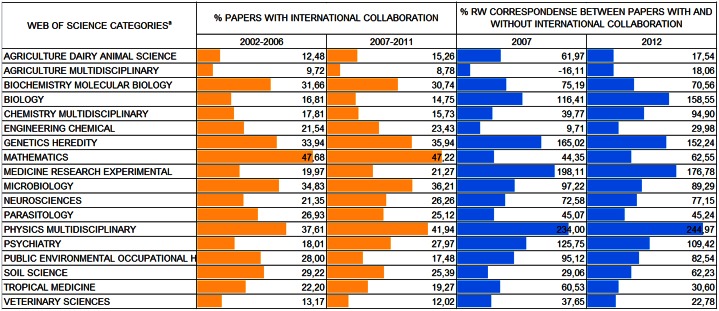
International collaboration and its influence on the impact of papers published in foreign journals. ^a^The “Social Sciences, Interdisciplinary” subfield was not included in the analysis due to its negligible quantity of papers with international collaboration published in foreign journals. [Data sourced from Thomson Reuters Web of Science].

We present below the production and impact results correlated with international scientific collaboration (Fig 3). The data are limited to works published in foreign journals, considering that few Brazilian papers with international collaboration were published in national journals.

We observed wide variation between the subfields with regard to the two aspects presented in Fig 3: the percentage of papers with international collaboration published in foreign journals and the percentage difference of the impact (Rw) between these papers and those written without international collaboration.

“Genetics & Heredity”, “Mathematics”, “Microbiology” and “Physics, Multidisciplinary” have the most international collaboration. “Agriculture, Dairy & Animal Science”, “Agriculture, Multidisciplinary” and “Veterinary Sciences” have the least international collaboration.

With regard to Rw values, we observed that the impact of nearly all subfields production is higher when the papers published in foreign journals are written with international collaboration (only one exception is observed, in “Agriculture, Multidisciplinary” in the first period). However, in some subfields, the difference in impact is quite small, as in “Agriculture, Multidisciplinary”, “Engineering, Chemical” and “Veterinary Sciences” in both periods.

At the other extreme are the subfields whose production impact is much greater when there is the participation of international researchers. This is the case of the subfields “Biology”, “Genetics & Heredity”, “Medicine, Research & Experimental”, “Physics, Multidisciplinary” and “Psychiatry”.

Closer analysis suggests the existence of collaborations at differing levels, which may explain the degree of influence of collaborations on the impact of production in specific subfields [21]. In general, the distinctive element occurs in studies that use a complex experimental structure, as observed in some clinical studies and genomic sequencing as well as in some researches that use particle accelerators and telescopes. In these cases, the number of authors involved in the papers far exceeds the average encountered in other publications in their respective areas, the hyperauthorship papers [22].

Very often, the hyperauthorship papers highlight a group name as the author. Over time, the bibliographic database began to specifically index this information to identify intellectual responsibility for eminently collaborative research [23]. In our sample, we identified papers with author-group information indexed in the WoS [24] only in the period between 2007 and 2011. A total of 433 papers were identified, 242 (56%) in “Physics, Multidisciplinary” and the others distributed among other 11 subfields.

**Fig 4:**
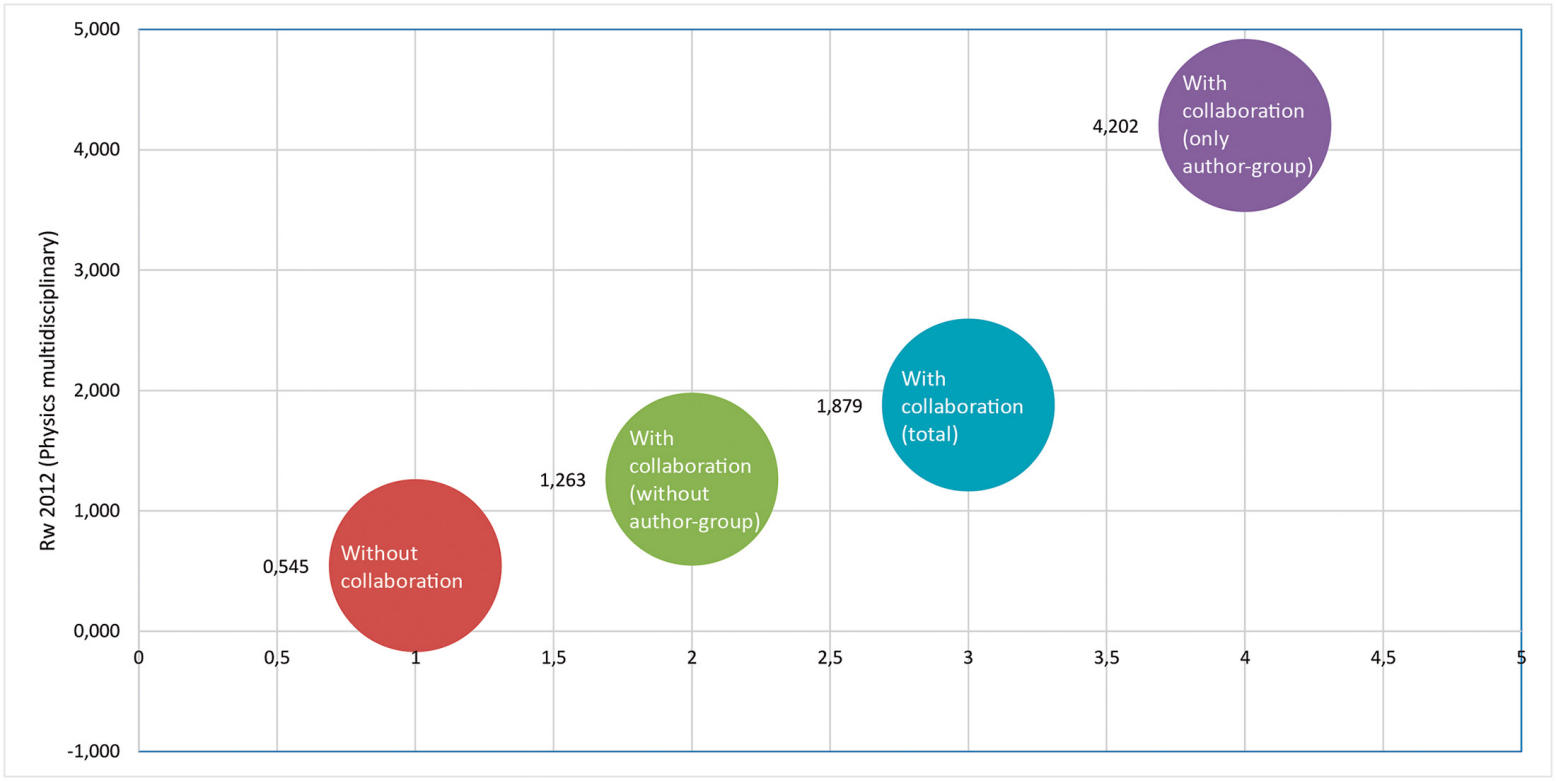
Rw 2012 of the “Physics, Multidisciplinary” subfield papers published in foreign journals by scientific collaboration category. [Data sourced from Thomson Reuters Web of Science].

In a more specific analysis, we calculated the Rw values with citations that appeared in 2012 against the author-group papers in these 11 subfields. In general, we verified only a small variation in the impact. However, a completely different configuration arises when we apply the same procedure to “Physics, Multidisciplinary”. In the Brazilian production in this subfield, we observed that author-group papers have a much greater impact than other papers with collaboration (Fig 4).

Although they represent approximately 21% of all papers published in foreign journals with international collaboration, the author-group papers account for nearly half of the citations received by that type of work in the “Physics, Multidisciplinary” sub-field. As shown in Fig 4, by excluding these papers to calculate Rw, we obtain a different perception of the impact of the Brazilian production in this sector. In our data, each author-group paper in “Physics, Multidisciplinary” is written on average by 1000 researchers and involves more than 20 countries.

## Discussion

The present study analyzed Brazil’s scientific production and its impact, taking into consideration certain issues that are particularly important to the least developed countries. Next, we discuss the most significant results, especially with regard to the role of national journals and to the methodological aspects of evaluating scientific publications by development agencies in Brazil.

Concerning the first aspect, we note that the role played by national journals in emerging countries is different from that in developed countries. Without commercial appeal, the so-called "emerging journals" [7] play a key role in developing skills at reporting and editing research results, as mentioned previously.

The importance of national journals as a communication channel for research results is observed by analyzing scientific production world rankings. Brazil is in top positions in 7 of the 19 subfields, among them, 6 subfields that predominantly publish in national journals.

In contrast, when analyzing Brazil’s position compared with countries that play a leading role in the scientific context, we realize that in addition to the measures given by the rankings, the country contributes with a negligible share of researchers who belong to the editorial boards of prestigious international journals [25,26]. In a sample analysis, García-Carpintero, Grenadian and Plaza observed that only 0.7% of researchers working in scientific publishing are Latin Americans [26]. This underrepresentation generates a gap in the cycle of Brazilian scientific research [7]: if, individually, authors act as communicators of the results from their specific research problems, collectively, editors contribute more broadly to scientific communication, enabling them to evaluate the results obtained by their peers and giving them perspective on the problems of research fields as a whole. For editors, the impact of a researcher’s work occurs beyond the research.

In Brazil, the gap in this cycle came to exist because the full development of a country’s science research is attained by training researchers to be able to realize and implement more comprehensive overviews of research fields in the editorial aspects of scientific publications. Today, however, this function is performed by Brazilian researchers in a restricted way nearly exclusively through the work of the editorial staffs of national journals [7]. Nevertheless, the impact of these editors’ performance in their respective research fields is limited because the visibility of national journals in the international community is also limited, even in subfields that publish papers written predominantly in English.

In essence, the limited visibility is because submissions to national journals are restricted to works written by Brazilian researchers themselves. The situation is aggravated because the contributions by Brazilians are generally submitted to national journals if they have not been accepted in other international journals or their authors predict that they will not be [27]. The results we obtained confirm the low visibility of the national journals and the significantly lower impact of the papers that Brazilian researchers publish in them. In most of the subfields, the impact of Brazil’s output published in national journals is less than half of the average global impact.

The other interesting question raised by the approach that we developed concerns the methodological procedures for measuring the impact of scientific production. The use of scientometric indicators by Brazilian funding agencies has become popular as a tool for evaluating scientific production and impact in areas in which production is predominantly reported in journals indexed by international databases. The adoption of this type of resource is a global trend because of the expansion of the S, T & I sector as a whole. Considering the difficulties faced so far in collecting citation data for specific works, the Garfield (impact) Factor (GF), which is annually published in the JCR, was for many years widely used to indicate the relevance of a contribution by the impact of the journal that published it. This impact assessment method has always received much criticism [16] and has been used less often with the evolution of information systems’ ability to aggregate data from citations to a list of papers.

However, regardless of the object—whether journal or paper—the need to normalize the impact measured by citation patterns in specific (sub)fields was the lesson learned from many years of using these indicators. Unfortunately, in Brazil, some sectors have not yet transposed this learning to evaluation practice. The Brazilian scientific community feels the effects and spreads complaints about the damage suffered by areas of lower impact at the time of the evaluations [28–30].

However, the normalized indicators we used showed that Brazilian papers that were published in foreign journals in 7 of the 19 subfields had greater impact than the world average. We emphasize that the direction is opposite because in these subfields, only one has a high standard of absolute impact; the others have reduced citation potential because they address themes that are considered regional or of an applied nature. The normalized indicator showed a different picture for the evaluation of these subfields.

Another point concerning evaluation methodological issues regards the studies that mobilize a large number of researchers, require sophisticated experimental structure and result in hyperauthorship papers. In addition to identifying the individual authors, these papers typically attribute authorship to a group given the cooperative nature of the research. Often, researchers involved in this type of research have much higher standards of productivity and scientific impact [31]. We have recently observed a proliferation of lists and rankings that aim to identify outstanding academic achievements. It is not uncommon that the top of these lists is almost exclusively composed of author-group studies. Less insightful interpretations induce the expectation that these rankings indicate the corresponding level of impact on scientific excellence, ignoring the distinction of these studies in scientometric terms.

The vast majority of scientific knowledge does not come from individual research that can sometimes engage thousands of researchers; this arrangement of scientific collaboration is as unusual as the productivity and the impact arisen from it. Our results highlight this distinction present in one of the subfields in which we identified a significant number of author-group papers that in turn had such superior impact that it altered the perception of performance in that area in the Brazilian context. Careful evaluation in this context requires recognizing the inadequacy of using absolute parameters and creating methodological approaches that allow for comparing these studies with others, maintaining the proportions of production and impact inherent to their respective realities.

Finally, we highlight that scientific performance analysis using quantitative indicators cannot capture the influence of psychosocial aspects in the citation selection criteria adopted by researchers. However, evidence on the effect of peripheral conditions in recognizing the achievements of papers published by Latin American researchers was obtained by Meneghini, Packer and Nassi-Calo [32] when they analyzed the relationship between the impact of the papers published in certain prestigious journals and the affiliations of their authors. In their study, the authors found that articles written by Latin American researchers tended to receive fewer citations than those published by authors in developed countries. That is, papers that address themes of international concern are good enough to be published in highly prestigious journals but are less cited. This existing dissociation in recognizing merit that occurs between the moment of a paper’s publication and its citation in the literature seems incomprehensible when we assume that the perception of a contribution’s quality relates exclusively to the pure technical ability demonstrated.

Currently, our research group is developing a study that adopts a qualitative approach to researchers’ perceptions of these immeasurable aspects by analyzing the products of scientific activity to complement the approach developed here.

## Supporting Information

S1 Appendix Layout and descriptions of the search strategies and the use of the WoS analysis resources for the composition of Sample 1. (DOCX)

S2 Appendix Layout and descriptions of the search strategies and analysis resources of the Web of Science utilized for the composition of Sample 2. (DOCX)
