# Supplementary material for: Brazilian Science between National and Foreign Journals: Methodology for Analyzing the Production and Impact in Emerging Scientific Communities
Source: PLoS One. 2016 May 12;11(5):e0155148. doi: 10.1371/journal.pone.0155148 (PMC4865143; doi:10.1371/journal.pone.0155148)
Supplement: S1 Appendix — (DOCX) [file pone.0155148.s001.docx]

**S1 Appendix: Layout and descriptions of the search strategies and the use of the WoS analysis resources for the composition of Sample 1.**

Fig. S1 presents the layout of the search strategy and describes how we utilize the analysis resources of the Web of Science for the Sample 1 composition. Tables S1-S6 complement the Fig. S1 with a full description of the search parameters by Web of Science field tags.

The Sample 1 data were collected from WoS on 12-23-2015, totalizing 252.270 papers. However, considering that WoS is updated almost every working day, the obtained figures will constantly depend on the day in which the database was accessed. Thus, when we collected the data on 3^rd,^ March 2016 we obtained 250,446 papers observing the criteria for compose the Sample 1. The reason for the difference between these results probably is due to the recategorization of the papers indexed in the WoS. This recategorization is mainly based on two parameters: “Document Type” or “WoS Subject Category”. It is important to emphasize that this reduction of the sample size does not change the conclusions drawn from our analysis.


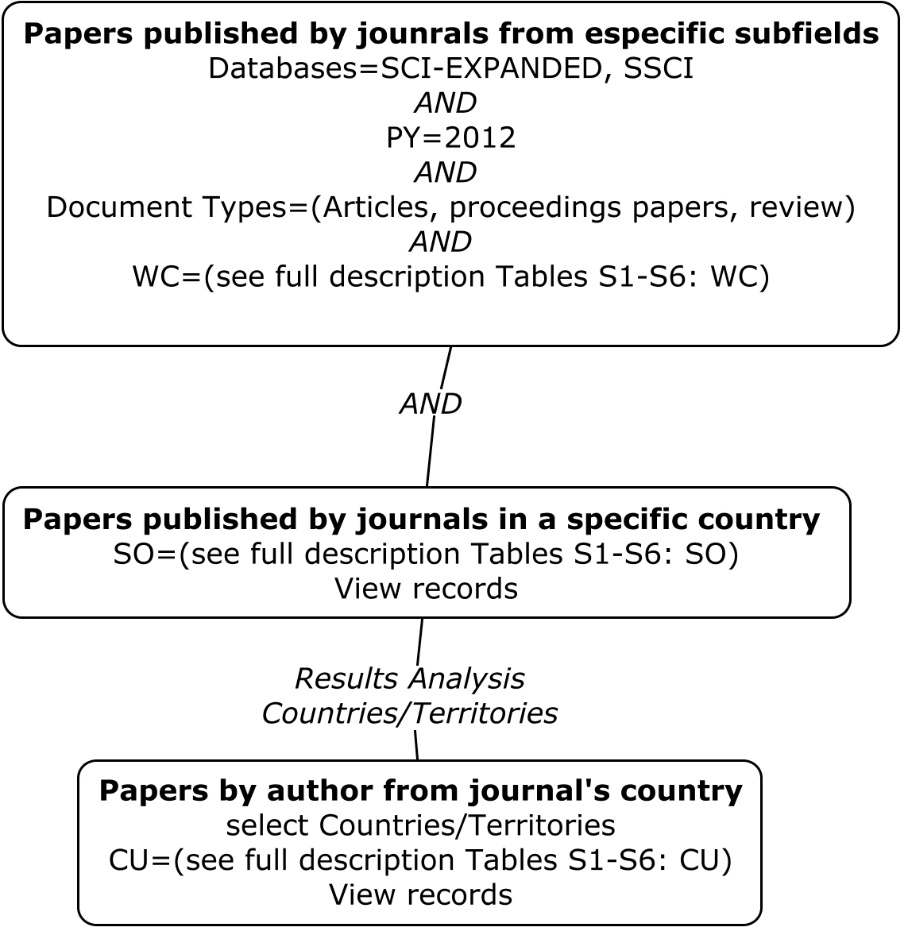


**Fig. S1. Layout of the search strategies and analysis resources utilized for the sample composition of papers published by journals from specific countries and its relation to the country affiliation of the authors.**

**Table S1. Full description of the search parameters related to the layout described in Fig. S1 by Web of Science Field Tags (journals publish in Brazil).**

| **Search parameters by Web of Science field tags** |
| --- |
|  |
| **WC** “Web of Science Category”  WC=(AGRICULTURE, DAIRY & ANIMAL SCIENCE OR AGRICULTURE, MULTIDISCIPLINARY OR BIOCHEMISTRY & MOLECULAR BIOLOGY OR BIOLOGY OR CHEMISTRY, MULTIDISCIPLINARY OR ENGINEERING, CHEMICAL OR GENETICS & HEREDITY OR MATHEMATICS OR MEDICINE, RESEARCH & EXPERIMENTAL OR MICROBIOLOGY OR NEUROSCIENCES OR PARASITOLOGY OR PHYSICS, MULTIDISCIPLINARY OR PSYCHIATRY OR PUBLIC, ENVIRONMENTAL & OCCUPATIONAL HEALTH OR SOCIAL SCIENCES, INTERDISCIPLINARY OR SOIL SCIENCE OR TROPICAL MEDICINE OR VETERINARY SCIENCES) |
| **SO** “Publication name”  SO=(Acta Scientiae Veterinariae OR Animal Reproduction OR ARQUIVO BRASILEIRO DE MEDICINA VETERINARIA E ZOOTECNIA OR ARQUIVOS DE NEURO-PSIQUIATRIA OR ARQUIVOS DE NEURO-PSIQUIATRIA OR Bioscience Journal OR Bioscience Journal OR BRAGANTIA OR BRAZILIAN ARCHIVES OF BIOLOGY AND TECHNOLOGY OR BRAZILIAN JOURNAL OF BIOLOGY OR BRAZILIAN JOURNAL OF CHEMICAL ENGINEERING OR BRAZILIAN JOURNAL OF MEDICAL AND BIOLOGICAL RESEARCH OR BRAZILIAN JOURNAL OF MEDICAL AND BIOLOGICAL RESEARCH OR BRAZILIAN JOURNAL OF MICROBIOLOGY OR BRAZILIAN JOURNAL OF PHYSICS OR Brazilian Journal of Poultry Science OR BULLETIN OF THE BRAZILIAN MATHEMATICAL SOCIETY OR Cadernos de Saude Publica OR Cadernos de Saude Publica OR Ciencia & Saude Coletiva OR CIENCIA E AGROTECNOLOGIA OR DADOS-REVISTA DE CIENCIAS SOCIAIS OR GENETICS AND MOLECULAR BIOLOGY OR GENETICS AND MOLECULAR BIOLOGY OR GENETICS AND MOLECULAR RESEARCH OR GENETICS AND MOLECULAR RESEARCH OR JOURNAL OF THE BRAZILIAN CHEMICAL SOCIETY OR JOURNAL OF VENOMOUS ANIMALS AND TOXINS INCLUDING TROPICAL DISEASES OR Medicina Veterinaria-Recife OR MEMORIAS DO INSTITUTO OSWALDO CRUZ OR MEMORIAS DO INSTITUTO OSWALDO CRUZ OR Movimento OR PESQUISA AGROPECUARIA BRASILEIRA OR PESQUISA VETERINARIA BRASILEIRA OR QUIMICA NOVA OR REVISTA BRASILEIRA DE CIENCIA DO SOLO OR REVISTA BRASILEIRA DE PARASITOLOGIA VETERINARIA OR REVISTA BRASILEIRA DE PARASITOLOGIA VETERINARIA OR REVISTA BRASILEIRA DE PSIQUIATRIA OR REVISTA BRASILEIRA DE PSIQUIATRIA OR REVISTA BRASILEIRA DE ZOOTECNIA-BRAZILIAN JOURNAL OF ANIMAL SCIENCE OR REVISTA BRASILEIRA DE ZOOTECNIA-BRAZILIAN JOURNAL OF ANIMAL SCIENCE OR Revista Ciencia Agronomica OR Revista da Sociedade Brasileira de Medicina Tropical OR Revista de Psiquiatria Clinica OR REVISTA DE SAUDE PUBLICA OR REVISTA DE SAUDE PUBLICA OR REVISTA DO INSTITUTO DE MEDICINA TROPICAL DE SAO PAULO OR Saude e Sociedade OR SCIENTIA AGRICOLA OR Semina-Ciencias Agrarias) |
| **CU** “COUNTRY”  CU=(BRAZIL) |

**Table S2. Full description of the parameters of search related to the described schemes in Fig. S1. by Web of Science field tags (journals published in England).**

| **Search parameters by Web of Science field tags** |
| --- |
|  |
| **WC** “Web of Science Category”  WC=(AGRICULTURE, DAIRY & ANIMAL SCIENCE OR AGRICULTURE, MULTIDISCIPLINARY OR BIOCHEMISTRY & MOLECULAR BIOLOGY OR BIOLOGY OR CHEMISTRY, MULTIDISCIPLINARY OR ENGINEERING, CHEMICAL OR GENETICS & HEREDITY OR MATHEMATICS OR MEDICINE, RESEARCH & EXPERIMENTAL OR MICROBIOLOGY OR NEUROSCIENCES OR PARASITOLOGY OR PHYSICS, MULTIDISCIPLINARY OR PSYCHIATRY OR PUBLIC, ENVIRONMENTAL & OCCUPATIONAL HEALTH OR SOCIAL SCIENCES, INTERDISCIPLINARY OR SOIL SCIENCE OR TROPICAL MEDICINE OR VETERINARY SCIENCES) |
| **SO** “Publication name”  SO=(ACCIDENT ANALYSIS AND PREVENTION OR ACCIDENT ANALYSIS AND PREVENTION OR ACTA AGRICULTURAE SCANDINAVICA SECTION A-ANIMAL SCIENCE OR ACTA PSYCHIATRICA SCANDINAVICA OR ACTA PSYCHIATRICA SCANDINAVICA OR Action Research OR ADAPTIVE BEHAVIOR OR ADDICTION OR ADDICTION OR ADDICTION BIOLOGY OR ADSORPTION SCIENCE & TECHNOLOGY OR Advances in Life Course Research OR ADVANCES IN THERAPY OR AGING & MENTAL HEALTH OR AGING & MENTAL HEALTH OR AIDS CARE-PSYCHOLOGICAL AND SOCIO-MEDICAL ASPECTS OF AIDS/HIV OR Algebraic and Geometric Topology OR Alzheimers Research & Therapy OR ANAEROBE OR Animal OR Animal OR ANIMAL GENETICS OR ANIMAL GENETICS OR ANIMAL HEALTH RESEARCH REVIEWS OR ANIMAL WELFARE OR Annals of Clinical Microbiology and Antimicrobials OR Annals of General Psychiatry OR Annals of General Psychiatry OR ANNALS OF HUMAN BIOLOGY OR ANNALS OF HUMAN BIOLOGY OR ANNALS OF HUMAN BIOLOGY OR ANNALS OF HUMAN GENETICS OR ANNALS OF OCCUPATIONAL HYGIENE OR ANTHROZOOS OR ANXIETY STRESS AND COPING OR ANXIETY STRESS AND COPING OR ANXIETY STRESS AND COPING OR APPLIED ENERGY OR Archives of Agronomy and Soil Science OR ARCHIVES OF ANIMAL NUTRITION OR ARCHIVES OF SUICIDE RESEARCH OR Asia-Pacific Journal of Chemical Engineering OR ASN Neuro OR ATLA-ALTERNATIVES TO LABORATORY ANIMALS OR AUSTRALIAN AND NEW ZEALAND JOURNAL OF PSYCHIATRY OR AUSTRALIAN AND NEW ZEALAND JOURNAL OF PSYCHIATRY OR AUSTRALIAN VETERINARY JOURNAL OR Avian Biology Research OR AVIAN PATHOLOGY OR Behavioral and Brain Functions OR BEHAVIORAL AND BRAIN SCIENCES OR BIOCATALYSIS AND BIOTRANSFORMATION OR BIOCHEMICAL JOURNAL OR BIOCHEMICAL SOCIETY TRANSACTIONS OR BIOCHEMICAL SYSTEMATICS AND ECOLOGY OR BIOLOGICAL REVIEWS OR BIOLOGICAL RHYTHM RESEARCH OR Biology Direct OR Biology Letters OR Biology of Sex Differences OR Biomarkers in Medicine OR BIOMEDICAL CHROMATOGRAPHY OR BIOMETRIKA OR BIOORGANIC & MEDICINAL CHEMISTRY OR BIOSYSTEMS ENGINEERING OR Biotechnology & Genetic Engineering Reviews OR BMC BIOCHEMISTRY OR BMC BIOLOGY OR BMC EVOLUTIONARY BIOLOGY OR BMC GENETICS OR BMC GENOMICS OR BMC International Health and Human Rights OR BMC Medical Genetics OR BMC Medical Genomics OR BMC MICROBIOLOGY OR BMC MOLECULAR BIOLOGY OR BMC NEUROSCIENCE OR BMC Psychiatry OR BMC PUBLIC HEALTH OR BMC Veterinary Research OR BMC Womens Health OR BRAIN OR BRAIN INJURY OR BRAIN PATHOLOGY OR Briefings in Functional Genomics OR BRITISH JOURNAL OF PSYCHIATRY OR BRITISH JOURNAL OF PSYCHIATRY OR BRITISH POULTRY SCIENCE OR BULLETIN OF SYMBOLIC LOGIC OR BULLETIN OF THE LONDON MATHEMATICAL SOCIETY OR Cancer Epidemiology OR CANCER GENE THERAPY OR CANCER GENE THERAPY OR CATTLE PRACTICE OR Cell and Bioscience OR CELL DEATH AND DIFFERENTIATION OR CELLULAR MICROBIOLOGY OR CHAOS SOLITONS & FRACTALS OR CHEMICAL COMMUNICATIONS OR CHEMICAL ENGINEERING RESEARCH & DESIGN OR Chemical Science OR CHEMICAL SOCIETY REVIEWS OR CHEMICAL SPECIATION AND BIOAVAILABILITY OR Chemistry Central Journal OR Child and Adolescent Mental Health OR Child and Adolescent Mental Health OR CHILDHOOD-A GLOBAL JOURNAL OF CHILD RESEARCH OR CLASSICAL AND QUANTUM GRAVITY OR Clinical and Investigative Medicine OR Clinical Lipidology OR CLINICAL MICROBIOLOGY AND INFECTION OR CLINICAL SCIENCE OR Clinical Trials OR Cognitive Neuropsychiatry OR Cognitive Neuropsychiatry OR Cognitive Neuroscience OR COLORATION TECHNOLOGY OR COMBUSTION THEORY AND MODELLING OR COMPARATIVE IMMUNOLOGY MICROBIOLOGY AND INFECTIOUS DISEASES OR COMPARATIVE IMMUNOLOGY MICROBIOLOGY AND INFECTIOUS DISEASES OR Complex Variables and Elliptic Equations OR COMPUTATIONAL BIOLOGY AND CHEMISTRY OR COMPUTERS AND ELECTRONICS IN AGRICULTURE OR CONTEMPORARY PHYSICS OR Criminal Behaviour and Mental Health OR Critical Public Health OR CRITICAL SOCIAL POLICY OR CRYOLETTERS OR CRYSTENGCOMM OR CURRENT ISSUES IN MOLECULAR BIOLOGY OR CURRENT MEDICAL RESEARCH AND OPINION OR CURRENT OPINION IN CHEMICAL BIOLOGY OR CURRENT OPINION IN LIPIDOLOGY OR CURRENT OPINION IN MICROBIOLOGY OR CURRENT OPINION IN NEUROBIOLOGY OR CURRENT OPINION IN STRUCTURAL BIOLOGY OR CYTOKINE OR CYTOKINE & GROWTH FACTOR REVIEWS OR CYTOTHERAPY OR Developmental Cognitive Neuroscience OR DISABILITY & SOCIETY OR DYES AND PIGMENTS OR eLife OR EMBO REPORTS OR Emerging Microbes & Infections OR Energy & Environmental Science OR Energy & Environmental Science OR Environmental Health OR ENVIRONMENTAL MICROBIOLOGY OR Environmental Microbiology Reports OR EPIDEMIOLOGY AND INFECTION OR Epidemiology and Psychiatric Sciences OR Epidemiology and Psychiatric Sciences OR Epigenetics & Chromatin OR Epigenomics OR EQUINE VETERINARY EDUCATION OR EQUINE VETERINARY JOURNAL OR ERGODIC THEORY AND DYNAMICAL SYSTEMS OR Essays in Biochemistry OR ETHNICITY & HEALTH OR EUROPEAN JOURNAL OF COMBINATORICS OR EUROPEAN JOURNAL OF CONTRACEPTION AND REPRODUCTIVE HEALTH CARE OR EUROPEAN JOURNAL OF HUMAN GENETICS OR EUROPEAN JOURNAL OF HUMAN GENETICS OR EUROPEAN JOURNAL OF NEUROLOGY OR EUROPEAN JOURNAL OF NEUROSCIENCE OR EUROPEAN JOURNAL OF PAIN OR EUROPEAN JOURNAL OF PHYSICS OR EUROPEAN JOURNAL OF PUBLIC HEALTH OR EUROPEAN JOURNAL OF PUBLIC HEALTH OR EUROPEAN JOURNAL OF SOIL SCIENCE OR Evidence & Policy OR EXPERIMENTAL AND MOLECULAR MEDICINE OR EXPERIMENTAL AND MOLECULAR MEDICINE OR EXPERIMENTAL BIOLOGY AND MEDICINE OR EXPERT OPINION ON BIOLOGICAL THERAPY OR Expert Opinion on Drug Metabolism & Toxicology OR EXPERT REVIEWS IN MOLECULAR MEDICINE OR EXPERT REVIEWS IN MOLECULAR MEDICINE OR FEBS Journal OR FEMS MICROBIOLOGY ECOLOGY OR FEMS MICROBIOLOGY LETTERS OR FEMS MICROBIOLOGY REVIEWS OR FEMS YEAST RESEARCH OR FILTRATION & SEPARATION OR FISH & SHELLFISH IMMUNOLOGY OR Food & Function OR FOOD AND BIOPRODUCTS PROCESSING OR FOOD MICROBIOLOGY OR FREE RADICAL RESEARCH OR FUEL OR Future Microbiology OR GENE THERAPY OR GENE THERAPY OR GENE THERAPY OR GENES AND IMMUNITY OR Genetics Research OR GENOME BIOLOGY OR Genome Biology and Evolution OR Genome Medicine OR Geobiology OR GLASGOW MATHEMATICAL JOURNAL OR Global Public Health OR Globalization and Health OR Globalization and Health OR Globalizations OR GREEN CHEMISTRY OR Green Chemistry Letters and Reviews OR Gut Pathogens OR HEALTH OR HEALTH & SOCIAL CARE IN THE COMMUNITY OR HEALTH EDUCATION JOURNAL OR HEALTH EDUCATION RESEARCH OR HEALTH EXPECTATIONS OR HEALTH EXPECTATIONS OR HEALTH PROMOTION INTERNATIONAL OR HEALTH RISK & SOCIETY OR HELICOBACTER OR HEREDITY OR HIGH PRESSURE RESEARCH OR HISTORY OF PSYCHIATRY OR Human Genomics OR HUMAN MOLECULAR GENETICS OR HUMAN MOLECULAR GENETICS OR HUMAN PSYCHOPHARMACOLOGY-CLINICAL AND EXPERIMENTAL OR HUMAN RELATIONS OR ILAR JOURNAL OR IN PRACTICE OR INDOOR AND BUILT ENVIRONMENT OR INJURY PREVENTION OR INJURY PREVENTION OR Innate Immunity OR Innate Immunity OR Innate Immunity OR INSECT BIOCHEMISTRY AND MOLECULAR BIOLOGY OR INSECT MOLECULAR BIOLOGY OR INTEGRAL TRANSFORMS AND SPECIAL FUNCTIONS OR INTERDISCIPLINARY SCIENCE REVIEWS OR Interface Focus OR International Health OR International Health OR International Journal for Equity in Health OR INTERNATIONAL JOURNAL FOR PARASITOLOGY OR International Journal for Parasitology-Drugs and Drug Resistance OR INTERNATIONAL JOURNAL OF ADHESION AND ADHESIVES OR International Journal of Agricultural Sustainability OR INTERNATIONAL JOURNAL OF BIOCHEMISTRY & CELL BIOLOGY OR INTERNATIONAL JOURNAL OF CLINICAL AND EXPERIMENTAL HYPNOSIS OR INTERNATIONAL JOURNAL OF DEVELOPMENTAL NEUROSCIENCE OR INTERNATIONAL JOURNAL OF ENVIRONMENTAL HEALTH RESEARCH OR INTERNATIONAL JOURNAL OF EPIDEMIOLOGY OR INTERNATIONAL JOURNAL OF GERIATRIC PSYCHIATRY OR INTERNATIONAL JOURNAL OF GERIATRIC PSYCHIATRY OR International Journal of Health Geographics OR INTERNATIONAL JOURNAL OF HEALTH PLANNING AND MANAGEMENT OR International Journal of Heritage Studies OR International Journal of Immunogenetics OR International Journal of Injury Control and Safety Promotion OR INTERNATIONAL JOURNAL OF LAW AND PSYCHIATRY OR International Journal of Mental Health Systems OR INTERNATIONAL JOURNAL OF METHODS IN PSYCHIATRIC RESEARCH OR INTERNATIONAL JOURNAL OF METHODS IN PSYCHIATRIC RESEARCH OR INTERNATIONAL JOURNAL OF NEUROPSYCHOPHARMACOLOGY OR INTERNATIONAL JOURNAL OF NEUROPSYCHOPHARMACOLOGY OR INTERNATIONAL JOURNAL OF NEUROSCIENCE OR International Journal of Qualitative Studies on Health and Well-Being OR INTERNATIONAL JOURNAL OF RADIATION BIOLOGY OR INTERNATIONAL JOURNAL OF SOCIAL PSYCHIATRY OR International Journal of Social Research Methodology OR INTERNATIONAL JOURNAL OF SYSTEMATIC AND EVOLUTIONARY MICROBIOLOGY OR INTERNATIONAL JOURNAL OF TECHNOLOGY ASSESSMENT IN HEALTH CARE OR INTERNATIONAL MATHEMATICS RESEARCH NOTICES OR INTERNATIONAL REVIEW OF PSYCHIATRY OR INVERTEBRATE NEUROSCIENCE OR ISME Journal OR JASSS-THE JOURNAL OF ARTIFICIAL SOCIETIES AND SOCIAL SIMULATION OR JOURNAL OF ADHESION OR JOURNAL OF AEROSOL SCIENCE OR JOURNAL OF AGRICULTURAL SCIENCE OR JOURNAL OF ANTIMICROBIAL CHEMOTHERAPY OR JOURNAL OF APPLIED MICROBIOLOGY OR JOURNAL OF BEHAVIOR THERAPY AND EXPERIMENTAL PSYCHIATRY OR JOURNAL OF BIOLOGICAL RHYTHMS OR JOURNAL OF BIOSOCIAL SCIENCE OR JOURNAL OF CELLULAR AND MOLECULAR MEDICINE OR JOURNAL OF CHEMICAL RESEARCH OR JOURNAL OF CHEMICAL TECHNOLOGY AND BIOTECHNOLOGY OR JOURNAL OF CHEMICAL TECHNOLOGY AND BIOTECHNOLOGY OR Journal of Cheminformatics OR JOURNAL OF CHILD PSYCHOLOGY AND PSYCHIATRY OR JOURNAL OF CHILD PSYCHOLOGY AND PSYCHIATRY OR JOURNAL OF COMPARATIVE PATHOLOGY OR JOURNAL OF DAIRY RESEARCH OR Journal of Developmental Origins of Health and Disease OR JOURNAL OF ELECTROMYOGRAPHY AND KINESIOLOGY OR JOURNAL OF ENZYME INHIBITION AND MEDICINAL CHEMISTRY OR JOURNAL OF EPIDEMIOLOGY AND COMMUNITY HEALTH OR JOURNAL OF EPIDEMIOLOGY AND COMMUNITY HEALTH OR JOURNAL OF EVOLUTIONARY BIOLOGY OR JOURNAL OF EXPERIMENTAL BIOLOGY OR Journal of Experimental Nanoscience OR JOURNAL OF FELINE MEDICINE AND SURGERY OR JOURNAL OF FISH DISEASES OR JOURNAL OF FOOD ENGINEERING OR JOURNAL OF FORENSIC PSYCHIATRY & PSYCHOLOGY OR JOURNAL OF GENDER STUDIES OR JOURNAL OF GENE MEDICINE OR JOURNAL OF GENE MEDICINE OR JOURNAL OF HELMINTHOLOGY OR Journal of Integrative Neuroscience OR JOURNAL OF INTELLECTUAL DISABILITY RESEARCH OR JOURNAL OF INTELLECTUAL DISABILITY RESEARCH OR JOURNAL OF INTERNATIONAL MEDICAL RESEARCH OR Journal of K-Theory OR JOURNAL OF LOSS PREVENTION IN THE PROCESS INDUSTRIES OR JOURNAL OF MEDICAL GENETICS OR JOURNAL OF MEDICAL MICROBIOLOGY OR JOURNAL OF MEDICAL SCREENING OR JOURNAL OF MICROENCAPSULATION OR JOURNAL OF MOLECULAR MICROBIOLOGY AND BIOTECHNOLOGY OR JOURNAL OF MOLECULAR RECOGNITION OR Journal of Natural Gas Science and Engineering OR Journal of Neural Engineering OR JOURNAL OF NEUROCHEMISTRY OR JOURNAL OF NEUROCHEMISTRY OR JOURNAL OF NEUROENDOCRINOLOGY OR Journal of NeuroEngineering and Rehabilitation OR JOURNAL OF NEUROGENETICS OR JOURNAL OF NEUROGENETICS OR Journal of Neuroinflammation OR JOURNAL OF NEUROLOGY NEUROSURGERY AND PSYCHIATRY OR Journal of Occupational Medicine and Toxicology OR JOURNAL OF PEPTIDE SCIENCE OR Journal of Physics A-Mathematical and Theoretical OR JOURNAL OF PHYSIOLOGY-LONDON OR JOURNAL OF PROCESS CONTROL OR Journal of Psychiatric and Mental Health Nursing OR Journal of Psychiatric and Mental Health Nursing OR JOURNAL OF PSYCHIATRIC RESEARCH OR JOURNAL OF PSYCHIATRIC RESEARCH OR JOURNAL OF PSYCHOPHARMACOLOGY OR JOURNAL OF PSYCHOPHARMACOLOGY OR JOURNAL OF PSYCHOPHARMACOLOGY OR JOURNAL OF PSYCHOSOMATIC RESEARCH OR JOURNAL OF PSYCHOSOMATIC RESEARCH OR JOURNAL OF PUBLIC HEALTH OR JOURNAL OF PUBLIC HEALTH OR JOURNAL OF PUBLIC HEALTH POLICY OR JOURNAL OF PUBLIC HEALTH POLICY OR JOURNAL OF RADIOLOGICAL PROTECTION OR JOURNAL OF RISK RESEARCH OR JOURNAL OF SLEEP RESEARCH OR JOURNAL OF SMALL ANIMAL PRACTICE OR JOURNAL OF STEROID BIOCHEMISTRY AND MOLECULAR BIOLOGY OR Journal of Sulfur Chemistry OR JOURNAL OF SYMBOLIC LOGIC OR Journal of the Institute of Mathematics of Jussieu OR JOURNAL OF THE LONDON MATHEMATICAL SOCIETY-SECOND SERIES OR JOURNAL OF THE SCIENCE OF FOOD AND AGRICULTURE OR JOURNAL OF THERMAL BIOLOGY OR Journal of Topology OR Journal of Translational Medicine OR JOURNAL OF TROPICAL PEDIATRICS OR Journal of Veterinary Cardiology OR JOURNAL OF VETERINARY PHARMACOLOGY AND THERAPEUTICS OR JOURNAL OF WATER AND HEALTH OR Journal of Youth Studies OR LAB ON A CHIP OR LABORATORY ANIMALS OR Lancet Global Health OR Lancet Global Health OR LAND DEGRADATION & DEVELOPMENT OR LEPROSY REVIEW OR LETTERS IN APPLIED MICROBIOLOGY OR LIFE SCIENCES OR LINEAR & MULTILINEAR ALGEBRA OR Lipids in Health and Disease OR LIQUID CRYSTALS OR LMS Journal of Computation and Mathematics OR LOGIC JOURNAL OF THE IGPL OR Lubrication Science OR LUMINESCENCE OR MAGNESIUM RESEARCH OR MAGNETIC RESONANCE IN CHEMISTRY OR MATHEMATICAL MEDICINE AND BIOLOGY-A JOURNAL OF THE IMA OR MATHEMATICAL PROCEEDINGS OF THE CAMBRIDGE PHILOSOPHICAL SOCIETY OR MATHEMATIKA OR MedChemComm OR MEDICAL AND VETERINARY ENTOMOLOGY OR MEDICAL MYCOLOGY OR Metallomics OR Methods in Microbiology OR MICROBIAL PATHOGENESIS OR MICROBIOLOGY-SGM OR MINERALS ENGINEERING OR Mitochondrial DNA OR MITOCHONDRION OR Mobile DNA OR MOLECULAR AND CELLULAR PROBES OR Molecular Autism OR Molecular Autism OR Molecular BioSystems OR Molecular Brain OR Molecular Cancer OR Molecular Cytogenetics OR MOLECULAR ECOLOGY OR Molecular Ecology Resources OR MOLECULAR IMMUNOLOGY OR MOLECULAR MICROBIOLOGY OR MOLECULAR MICROBIOLOGY OR Molecular Neurodegeneration OR Molecular Pain OR MOLECULAR PSYCHIATRY OR MOLECULAR PSYCHIATRY OR MOLECULAR PSYCHIATRY OR MUTAGENESIS OR Nano Today OR Nanoscale OR NATURAL PRODUCT REPORTS OR Nature Chemistry OR Nature Physics OR NATURE REVIEWS GENETICS OR NATURE REVIEWS MICROBIOLOGY OR NATURE REVIEWS NEUROSCIENCE OR NETWORK-COMPUTATION IN NEURAL SYSTEMS OR Neural Development OR NEURAL NETWORKS OR NEUROBIOLOGY OF AGING OR NEUROBIOLOGY OF DISEASE OR NEUROCHEMISTRY INTERNATIONAL OR NEUROCHEMISTRY INTERNATIONAL OR NEUROGASTROENTEROLOGY AND MOTILITY OR NEUROPATHOLOGY AND APPLIED NEUROBIOLOGY OR NEUROPHARMACOLOGY OR Neuropsychiatry OR Neuropsychiatry OR NEUROPSYCHOLOGIA OR NEUROPSYCHOLOGICAL REHABILITATION OR NEUROPSYCHOPHARMACOLOGY OR NEUROPSYCHOPHARMACOLOGY OR NEUROPSYCHOPHARMACOLOGY OR NEUROPSYCHOPHARMACOLOGY OR NEUROSCIENCE OR NEUROSCIENCE AND BIOBEHAVIORAL REVIEWS OR NEUROSCIENCE AND BIOBEHAVIORAL REVIEWS OR NEUROTOXICOLOGY AND TERATOLOGY OR NEW GENETICS AND SOCIETY OR NEW JOURNAL OF CHEMISTRY OR NEW JOURNAL OF PHYSICS OR NEW LEFT REVIEW OR NICOTINE & TOBACCO RESEARCH OR NICOTINE & TOBACCO RESEARCH OR Noise & Health OR NONLINEAR ANALYSIS-THEORY METHODS & APPLICATIONS OR NUCLEIC ACIDS RESEARCH OR NUMERICAL LINEAR ALGEBRA WITH APPLICATIONS OR NUTRITIONAL NEUROSCIENCE OR OCCUPATIONAL AND ENVIRONMENTAL MEDICINE OR OCCUPATIONAL MEDICINE-OXFORD OR ONCOGENE OR ONCOGENE OR Open Biology OR Orphanet Journal of Rare Diseases OR Orphanet Journal of Rare Diseases OR OUTLOOK ON AGRICULTURE OR PAEDIATRIC AND PERINATAL EPIDEMIOLOGY OR PALLIATIVE MEDICINE OR PARASITE IMMUNOLOGY OR Parasites & Vectors OR PARASITOLOGY OR Pathogens and Disease OR Pathogens and Global Health OR Pathogens and Global Health OR Pathogens and Global Health OR Personality and Mental Health OR Perspectives in Public Health OR PHARMACOLOGY BIOCHEMISTRY AND BEHAVIOR OR PHILOSOPHICAL TRANSACTIONS OF THE ROYAL SOCIETY B-BIOLOGICAL SCIENCES OR PHOTOCHEMICAL & PHOTOBIOLOGICAL SCIENCES OR PHYSICS WORLD OR PHYTOCHEMISTRY OR Pigment & Resin Technology OR Population Health Metrics OR PRENATAL DIAGNOSIS OR PROCEEDINGS OF THE EDINBURGH MATHEMATICAL SOCIETY OR PROCEEDINGS OF THE LONDON MATHEMATICAL SOCIETY OR PROCEEDINGS OF THE ROYAL SOCIETY B-BIOLOGICAL SCIENCES OR PROCESS BIOCHEMISTRY OR PROCESS BIOCHEMISTRY OR PROCESS SAFETY AND ENVIRONMENTAL PROTECTION OR PROGRESS IN BIOPHYSICS & MOLECULAR BIOLOGY OR PROGRESS IN ENERGY AND COMBUSTION SCIENCE OR PROGRESS IN LIPID RESEARCH OR PROGRESS IN NEUROBIOLOGY OR PROGRESS IN NEURO-PSYCHOPHARMACOLOGY & BIOLOGICAL PSYCHIATRY OR PROGRESS IN NEURO-PSYCHOPHARMACOLOGY & BIOLOGICAL PSYCHIATRY OR Progress of Theoretical and Experimental Physics OR PROTEIN ENGINEERING DESIGN & SELECTION OR PSYCHOLOGY & HEALTH OR PSYCHOLOGY AND PSYCHOTHERAPY-THEORY RESEARCH AND PRACTICE OR PSYCHOLOGY AND PSYCHOTHERAPY-THEORY RESEARCH AND PRACTICE OR Psychology Health & Medicine OR Psychology Health & Medicine OR PSYCHONEUROENDOCRINOLOGY OR PSYCHONEUROENDOCRINOLOGY OR Psychosis-Psychological Social and Integrative Approaches OR PUBLIC HEALTH OR PUBLIC HEALTH OR Public Health Ethics OR Public Health Ethics OR PUBLIC HEALTH NUTRITION OR PUBLIC OPINION QUARTERLY OR PURE AND APPLIED CHEMISTRY OR QUARTERLY JOURNAL OF MATHEMATICS OR RACE & CLASS OR RADIATION PROTECTION DOSIMETRY OR REDOX REPORT OR RENEWABLE AGRICULTURE AND FOOD SYSTEMS OR REPORTS ON PROGRESS IN PHYSICS OR Reproductive Health OR Reproductive Health OR REPRODUCTIVE HEALTH MATTERS OR Research in Autism Spectrum Disorders OR RESEARCH IN VETERINARY SCIENCE OR REVIEWS IN CHEMICAL ENGINEERING OR REVIEWS IN THE NEUROSCIENCES OR Risk Management-An International Journal OR RSC Advances OR SAR AND QSAR IN ENVIRONMENTAL RESEARCH OR SBORNIK MATHEMATICS OR SEIZURE-EUROPEAN JOURNAL OF EPILEPSY OR SLEEP MEDICINE REVIEWS OR SOCIAL & LEGAL STUDIES OR Social Cognitive and Affective Neuroscience OR Social Neuroscience OR SOCIAL PHILOSOPHY & POLICY OR SOCIAL SCIENCE & MEDICINE OR SOCIAL SCIENCE & MEDICINE OR Social Science Japan Journal OR SOCIOLOGY OF HEALTH & ILLNESS OR Soft Matter OR SOIL BIOLOGY & BIOCHEMISTRY OR SOIL USE AND MANAGEMENT OR SOMATOSENSORY AND MOTOR RESEARCH OR SPACE POLICY OR STATISTICS IN MEDICINE OR STATISTICS IN MEDICINE OR Stem Cell Research & Therapy OR STRESS AND HEALTH OR STRESS AND HEALTH OR STRUCTURE OR STUDIES IN HISTORY AND PHILOSOPHY OF MODERN PHYSICS OR SUPRAMOLECULAR CHEMISTRY OR SURFACE COATINGS INTERNATIONAL OR SYSTEMS RESEARCH AND BEHAVIORAL SCIENCE OR TIME & SOCIETY OR TOBACCO CONTROL OR TOBACCO CONTROL OR Tobacco Induced Diseases OR Tobacco Induced Diseases OR TRANSACTIONS OF THE ROYAL SOCIETY OF TROPICAL MEDICINE AND HYGIENE OR TRANSACTIONS OF THE ROYAL SOCIETY OF TROPICAL MEDICINE AND HYGIENE OR Transcultural Psychiatry OR Travel Medicine and Infectious Disease OR TRENDS IN COGNITIVE SCIENCES OR TRENDS IN ECOLOGY & EVOLUTION OR TRENDS IN MOLECULAR MEDICINE OR TRENDS IN MOLECULAR MEDICINE OR Trials OR TROPICAL DOCTOR OR TROPICAL DOCTOR OR TROPICAL MEDICINE & INTERNATIONAL HEALTH OR TROPICAL MEDICINE & INTERNATIONAL HEALTH OR TUBERCULOSIS OR ULTRASONICS SONOCHEMISTRY OR VACCINE OR VETERINARY ANAESTHESIA AND ANALGESIA OR Veterinary and Comparative Oncology OR VETERINARY DERMATOLOGY OR VETERINARY JOURNAL OR VETERINARY QUARTERLY OR VETERINARY RECORD OR VISION RESEARCH OR Waves in Random and Complex Media OR Wiley Interdisciplinary Reviews-Computational Molecular Science OR WORLD JOURNAL OF BIOLOGICAL PSYCHIATRY OR WORLDS POULTRY SCIENCE JOURNAL OR YEAST OR YEAST OR Young) |
| **CU** “COUNTRY”  CU =( ENGLAND) |

**Table S3. Full description of the parameters of search related to the described schemes in Fig. S1. by Web of Science field tags (journals published in Netherlands).**

| **Search parameters by Web of Science field tags** |
| --- |
|  |
| **WC** “Web of Science Category”  WC=(AGRICULTURE, DAIRY & ANIMAL SCIENCE OR AGRICULTURE, MULTIDISCIPLINARY OR BIOCHEMISTRY & MOLECULAR BIOLOGY OR BIOLOGY OR CHEMISTRY, MULTIDISCIPLINARY OR ENGINEERING, CHEMICAL OR GENETICS & HEREDITY OR MATHEMATICS OR MEDICINE, RESEARCH & EXPERIMENTAL OR MICROBIOLOGY OR NEUROSCIENCES OR PARASITOLOGY OR PHYSICS, MULTIDISCIPLINARY OR PSYCHIATRY OR PUBLIC, ENVIRONMENTAL & OCCUPATIONAL HEALTH OR SOCIAL SCIENCES, INTERDISCIPLINARY OR SOIL SCIENCE OR TROPICAL MEDICINE OR VETERINARY SCIENCES) |
| **SO** “Publication name”  SO=( ACTA TROPICA OR ACTA TROPICA OR ADSORPTION-JOURNAL OF THE INTERNATIONAL ADSORPTION SOCIETY OR AEROBIOLOGIA OR AGRICULTURAL SYSTEMS OR AGRICULTURE AND HUMAN VALUES OR AGRICULTURE ECOSYSTEMS & ENVIRONMENT OR ALGEBRAS AND REPRESENTATION THEORY OR ANIMAL FEED SCIENCE AND TECHNOLOGY OR ANIMAL REPRODUCTION SCIENCE OR ANNALS OF GLOBAL ANALYSIS AND GEOMETRY OR ANNALS OF PURE AND APPLIED LOGIC OR ANTONIE VAN LEEUWENHOEK INTERNATIONAL JOURNAL OF GENERAL AND MOLECULAR MICROBIOLOGY OR APPLIED ANIMAL BEHAVIOUR SCIENCE OR APPLIED ANIMAL BEHAVIOUR SCIENCE OR APPLIED CATALYSIS B-ENVIRONMENTAL OR APPLIED CATEGORICAL STRUCTURES OR Applied Research in Quality of Life OR APPLIED SOIL ECOLOGY OR Asian Journal of Social Science OR AUTONOMIC NEUROSCIENCE-BASIC & CLINICAL OR BEHAVIOURAL BRAIN RESEARCH OR BEHAVIOURAL BRAIN RESEARCH OR Beneficial Microbes OR BIOCHEMICAL ENGINEERING JOURNAL OR BIOCHIMICA ET BIOPHYSICA ACTA-BIOENERGETICS OR BIOCHIMICA ET BIOPHYSICA ACTA-BIOMEMBRANES OR Biochimica et Biophysica Acta-Gene Regulatory Mechanisms OR BIOCHIMICA ET BIOPHYSICA ACTA-GENERAL SUBJECTS OR BIOCHIMICA ET BIOPHYSICA ACTA-MOLECULAR AND CELL BIOLOGY OF LIPIDS OR BIOCHIMICA ET BIOPHYSICA ACTA-MOLECULAR BASIS OF DISEASE OR BIOCHIMICA ET BIOPHYSICA ACTA-MOLECULAR CELL RESEARCH OR BIOCHIMICA ET BIOPHYSICA ACTA-PROTEINS AND PROTEOMICS OR BIOCHIMICA ET BIOPHYSICA ACTA-REVIEWS ON CANCER OR BIOELECTROCHEMISTRY OR BIOELECTROCHEMISTRY OR BIOMETALS OR BIOPHYSICAL CHEMISTRY OR Body Image OR BRAIN RESEARCH OR CANCER CAUSES & CONTROL OR CARBOHYDRATE RESEARCH OR CATALYSIS TODAY OR CHEMICO-BIOLOGICAL INTERACTIONS OR CHEMISTRY AND PHYSICS OF LIPIDS OR Child Indicators Research OR CHROMOSOME RESEARCH OR CHROMOSOME RESEARCH OR Cognitive Neurodynamics OR Cognitive Systems Research OR COMMUNITY MENTAL HEALTH JOURNAL OR COMMUNITY MENTAL HEALTH JOURNAL OR COMPOSITIO MATHEMATICA OR COMPUTATIONAL GEOMETRY-THEORY AND APPLICATIONS OR CONSERVATION GENETICS OR Conservation Genetics Resources OR CRIME LAW AND SOCIAL CHANGE OR CULTURE MEDICINE AND PSYCHIATRY OR DESALINATION OR DIFFERENTIAL GEOMETRY AND ITS APPLICATIONS OR DISCRETE MATHEMATICS OR DISEASE MARKERS OR DISEASE MARKERS OR DNA REPAIR OR Eating Behaviors OR Economics & Human Biology OR Economics & Human Biology OR ENVIRONMENTAL GEOCHEMISTRY AND HEALTH OR EUROPEAN JOURNAL OF EPIDEMIOLOGY OR European Journal of Medical Genetics OR EUROPEAN NEUROPSYCHOPHARMACOLOGY OR EUROPEAN NEUROPSYCHOPHARMACOLOGY OR EVOLUTIONARY ECOLOGY OR Familial Cancer OR FEBS LETTERS OR FISH PHYSIOLOGY AND BIOCHEMISTRY OR FLUID PHASE EQUILIBRIA OR FUEL PROCESSING TECHNOLOGY OR GENE OR GENE EXPRESSION PATTERNS OR GENETICA OR GEODERMA OR GEOMETRIAE DEDICATA OR GLYCOCONJUGATE JOURNAL OR GROUP DECISION AND NEGOTIATION OR HEARING RESEARCH OR HUMAN MOVEMENT SCIENCE OR INDAGATIONES MATHEMATICAE-NEW SERIES OR INTERNATIONAL JOURNAL OF ANTIMICROBIAL AGENTS OR INTERNATIONAL JOURNAL OF BIOLOGICAL MACROMOLECULES OR INTERNATIONAL JOURNAL OF FOOD MICROBIOLOGY OR INTERNATIONAL JOURNAL OF MINERAL PROCESSING OR INTERNATIONAL JOURNAL OF PSYCHOPHYSIOLOGY OR JOURNAL OF ADHESION SCIENCE AND TECHNOLOGY OR JOURNAL OF AFFECTIVE DISORDERS OR JOURNAL OF AFFECTIVE DISORDERS OR JOURNAL OF AGRICULTURAL & ENVIRONMENTAL ETHICS OR JOURNAL OF ALZHEIMERS DISEASE OR JOURNAL OF BIOMOLECULAR NMR OR JOURNAL OF CHEMICAL ECOLOGY OR JOURNAL OF CHEMICAL NEUROANATOMY OR JOURNAL OF CHEMICAL NEUROANATOMY OR JOURNAL OF COMPUTER-AIDED MOLECULAR DESIGN OR JOURNAL OF CONTROLLED RELEASE OR Journal of Happiness Studies OR JOURNAL OF INCLUSION PHENOMENA AND MACROCYCLIC CHEMISTRY OR JOURNAL OF INHERITED METABOLIC DISEASE OR JOURNAL OF MEMBRANE SCIENCE OR JOURNAL OF MICROBIOLOGICAL METHODS OR JOURNAL OF MOLECULAR CATALYSIS B-ENZYMATIC OR JOURNAL OF NANOPARTICLE RESEARCH OR JOURNAL OF NEUROIMMUNOLOGY OR JOURNAL OF NEUROSCIENCE METHODS OR Journal of Obsessive-Compulsive and Related Disorders OR Journal of Obsessive-Compulsive and Related Disorders OR Journal of Parkinsons Disease OR JOURNAL OF PSYCHOSOMATIC OBSTETRICS AND GYNECOLOGY OR JOURNAL OF PURE AND APPLIED ALGEBRA OR JOURNAL OF SUPERCRITICAL FLUIDS OR JOURNAL OF THE AMERICAN ACADEMY OF CHILD AND ADOLESCENT PSYCHIATRY OR JOURNAL OF THE AMERICAN ACADEMY OF CHILD AND ADOLESCENT PSYCHIATRY OR JOURNAL OF THE HISTORY OF BIOLOGY OR JOURNAL OF THE NEUROLOGICAL SCIENCES OR Journal of Veterinary Behavior-Clinical Applications and Research OR Livestock Science OR MAIN GROUP CHEMISTRY OR MARINE CHEMISTRY OR Marine Genomics OR MATRIX BIOLOGY OR MINERVA OR MOLECULAR AND BIOCHEMICAL PARASITOLOGY OR MOLECULAR AND BIOCHEMICAL PARASITOLOGY OR MOLECULAR ASPECTS OF MEDICINE OR MOLECULAR ASPECTS OF MEDICINE OR MOLECULAR BIOLOGY REPORTS OR MOLECULAR BREEDING OR MOLECULAR DIVERSITY OR MUTATION RESEARCH-FUNDAMENTAL AND MOLECULAR MECHANISMS OF MUTAGENESIS OR MUTATION RESEARCH-GENETIC TOXICOLOGY AND ENVIRONMENTAL MUTAGENESIS OR MUTATION RESEARCH-REVIEWS IN MUTATION RESEARCH OR Nanomedicine-Nanotechnology Biology and Medicine OR NEUROSCIENCE LETTERS OR NEUROSCIENCE RESEARCH OR NEUROTOXICOLOGY OR NJAS-WAGENINGEN JOURNAL OF LIFE SCIENCES OR NUTRIENT CYCLING IN AGROECOSYSTEMS OR ORDER-A JOURNAL ON THE THEORY OF ORDERED SETS AND ITS APPLICATIONS OR ORIGINS OF LIFE AND EVOLUTION OF BIOSPHERES OR PAIN OR PHYSICA A-STATISTICAL MECHANICS AND ITS APPLICATIONS OR PHYSICA D-NONLINEAR PHENOMENA OR PHYSICS LETTERS A OR PHYSICS LETTERS B OR Physics of Life Reviews OR PHYSICS REPORTS-REVIEW SECTION OF PHYSICS LETTERS OR Phytochemistry Letters OR PLANT AND SOIL OR PLANT MOLECULAR BIOLOGY OR POLICY SCIENCES OR POSITIVITY OR POTENTIAL ANALYSIS OR PRECISION AGRICULTURE OR PREVENTIVE VETERINARY MEDICINE OR Progress in Brain Research OR PSYCHIATRY RESEARCH OR PSYCHIATRY RESEARCH OR PSYCHIATRY RESEARCH-NEUROIMAGING OR Purinergic Signalling OR QUALITY & QUANTITY OR QUALITY OF LIFE RESEARCH OR QUALITY OF LIFE RESEARCH OR RAMANUJAN JOURNAL OR REACTIVE & FUNCTIONAL POLYMERS OR RESEARCH IN MICROBIOLOGY OR RESEARCH ON CHEMICAL INTERMEDIATES OR RESTORATIVE NEUROLOGY AND NEUROSCIENCE OR SCHIZOPHRENIA RESEARCH OR SCHIZOPHRENIA RESEARCH OR SEPARATION AND PURIFICATION TECHNOLOGY OR SMALL RUMINANT RESEARCH OR SOCIAL INDICATORS RESEARCH OR SOCIAL SCIENCE INFORMATION SUR LES SCIENCES SOCIALES OR SOCIAL SCIENCE JOURNAL OR SOCIETY & ANIMALS OR SOIL & TILLAGE RESEARCH OR STRESS-THE INTERNATIONAL JOURNAL ON THE BIOLOGY OF STRESS OR SYMBIOSIS OR SYSTEMATIC PARASITOLOGY OR TIJDSCHRIFT VOOR DIERGENEESKUNDE OR TOPOLOGY AND ITS APPLICATIONS OR TRANSGENIC RESEARCH OR TRANSPORT IN POROUS MEDIA OR TRENDS IN BIOCHEMICAL SCIENCES OR TRENDS IN GENETICS OR TRENDS IN NEUROSCIENCES OR TRENDS IN NEUROSCIENCES OR TROPICAL ANIMAL HEALTH AND PRODUCTION OR TROPICAL ANIMAL HEALTH AND PRODUCTION OR VETERINARY IMMUNOLOGY AND IMMUNOPATHOLOGY OR VETERINARY MICROBIOLOGY OR VETERINARY MICROBIOLOGY OR VETERINARY PARASITOLOGY OR VETERINARY PARASITOLOGY OR VETERINARY RESEARCH COMMUNICATIONS OR WORK-A Journal of Prevention Assessment & Rehabilitation) |
| **CU** “COUNTRY”  CU =( NETHERLANDS) |

**Table S4. Full description of the parameters of search related to the described schemes in Fig. S1. by Web of Science field tags (journals published in China).**

| **Search parameters by Web of Science field tags** |
| --- |
|  |
| **WC** “Web of Science Category”  WC=(AGRICULTURE, DAIRY & ANIMAL SCIENCE OR AGRICULTURE, MULTIDISCIPLINARY OR BIOCHEMISTRY & MOLECULAR BIOLOGY OR BIOLOGY OR CHEMISTRY, MULTIDISCIPLINARY OR ENGINEERING, CHEMICAL OR GENETICS & HEREDITY OR MATHEMATICS OR MEDICINE, RESEARCH & EXPERIMENTAL OR MICROBIOLOGY OR NEUROSCIENCES OR PARASITOLOGY OR PHYSICS, MULTIDISCIPLINARY OR PSYCHIATRY OR PUBLIC, ENVIRONMENTAL & OCCUPATIONAL HEALTH OR SOCIAL SCIENCES, INTERDISCIPLINARY OR SOIL SCIENCE OR TROPICAL MEDICINE OR VETERINARY SCIENCES) |
| **SO** “Publication name”  SO=( ACTA BIOCHIMICA ET BIOPHYSICA SINICA OR ACTA CHIMICA SINICA OR ACTA MATHEMATICA SCIENTIA OR ACTA MATHEMATICA SINICA-ENGLISH SERIES OR ACTA PHARMACOLOGICA SINICA OR ACTA PHYSICA SINICA OR ALGEBRA COLLOQUIUM OR Asian Pacific Journal of Tropical Medicine OR Asian Pacific Journal of Tropical Medicine OR BIOMEDICAL AND ENVIRONMENTAL SCIENCES OR CHEMICAL JOURNAL OF CHINESE UNIVERSITIES-CHINESE OR CHEMICAL RESEARCH IN CHINESE UNIVERSITIES OR China Petroleum Processing & Petrochemical Technology OR CHINESE ANNALS OF MATHEMATICS SERIES B OR CHINESE CHEMICAL LETTERS OR CHINESE JOURNAL OF CATALYSIS OR CHINESE JOURNAL OF CHEMICAL ENGINEERING OR CHINESE JOURNAL OF CHEMISTRY OR Chinese Physics B OR CHINESE PHYSICS LETTERS OR CNS Neuroscience & Therapeutics OR COMMUNICATIONS IN THEORETICAL PHYSICS OR Frontiers of Mathematics in China OR Frontiers of Physics OR Journal of Animal Science and Biotechnology OR JOURNAL OF COMPUTATIONAL MATHEMATICS OR Journal of Energy Chemistry OR Journal of Genetics and Genomics OR Journal of Genetics and Genomics OR Journal of Huazhong University of Science and Technology-Medical Sciences OR Journal of Integrative Agriculture OR Journal of Integrative Plant Biology OR Journal of Zhejiang University-SCIENCE B OR Journal of Zhejiang University-SCIENCE B OR Molecular Plant OR Neural Regeneration Research OR Neuroscience Bulletin OR Numerical Mathematics-Theory Methods and Applications OR Particuology OR PEDOSPHERE OR PROGRESS IN BIOCHEMISTRY AND BIOPHYSICS OR PROGRESS IN CHEMISTRY OR Science China-Chemistry OR Science China-Life Sciences OR Science China-Mathematics OR Science China-Physics Mechanics & Astronomy) |
| **CU** “COUNTRY”  CU =( PEOPLES R CHINA) |

**Table S5. Full description of the parameters of search related to the described schemes in Fig. S1. by Web of Science field tags (journals published in Russia).**

| **Search parameters by Web of Science field tags** |
| --- |
|  |
| **WC** “Web of Science Category”  WC=(AGRICULTURE, DAIRY & ANIMAL SCIENCE OR AGRICULTURE, MULTIDISCIPLINARY OR BIOCHEMISTRY & MOLECULAR BIOLOGY OR BIOLOGY OR CHEMISTRY, MULTIDISCIPLINARY OR ENGINEERING, CHEMICAL OR GENETICS & HEREDITY OR MATHEMATICS OR MEDICINE, RESEARCH & EXPERIMENTAL OR MICROBIOLOGY OR NEUROSCIENCES OR PARASITOLOGY OR PHYSICS, MULTIDISCIPLINARY OR PSYCHIATRY OR PUBLIC, ENVIRONMENTAL & OCCUPATIONAL HEALTH OR SOCIAL SCIENCES, INTERDISCIPLINARY OR SOIL SCIENCE OR TROPICAL MEDICINE OR VETERINARY SCIENCES) |
| **SO** “Publication name”  SO=( Algebra and Logic OR APPLIED BIOCHEMISTRY AND MICROBIOLOGY OR BIOCHEMISTRY-MOSCOW OR BULLETIN OF EXPERIMENTAL BIOLOGY AND MEDICINE OR Bulletin of the Lebedev Physics Institute OR CHEMISTRY AND TECHNOLOGY OF FUELS AND OILS OR COMBUSTION EXPLOSION AND SHOCK WAVES OR Comparative Cytogenetics OR Cytology and Genetics OR DIFFERENTIAL EQUATIONS OR Doklady Biochemistry and Biophysics OR DOKLADY MATHEMATICS OR DOKLADY PHYSICS OR EURASIAN SOIL SCIENCE OR FIBRE CHEMISTRY OR FUNCTIONAL ANALYSIS AND ITS APPLICATIONS OR IZVESTIYA MATHEMATICS OR JETP LETTERS OR JOURNAL OF EVOLUTIONARY BIOCHEMISTRY AND PHYSIOLOGY OR JOURNAL OF EXPERIMENTAL AND THEORETICAL PHYSICS OR Macroheterocycles OR MATHEMATICAL NOTES OR MENDELEEV COMMUNICATIONS OR MICROBIOLOGY OR MOLECULAR BIOLOGY OR Moscow Mathematical Journal OR Moscow University Physics Bulletin OR Neurochemical Journal OR PETROLEUM CHEMISTRY OR Physics of Wave Phenomena OR PHYSICS-USPEKHI OR Proceedings of the Steklov Institute of Mathematics OR RUSSIAN CHEMICAL BULLETIN OR RUSSIAN CHEMICAL REVIEWS OR RUSSIAN JOURNAL OF BIOORGANIC CHEMISTRY OR RUSSIAN JOURNAL OF GENERAL CHEMISTRY OR RUSSIAN JOURNAL OF GENETICS OR RUSSIAN MATHEMATICAL SURVEYS OR Russian Physics Journal OR SIBERIAN MATHEMATICAL JOURNAL OR Solid Fuel Chemistry OR Solid Fuel Chemistry OR St Petersburg Mathematical Journal OR THEORETICAL AND MATHEMATICAL PHYSICS OR THEORETICAL FOUNDATIONS OF CHEMICAL ENGINEERING OR ZHURNAL OBSHCHEI BIOLOGII OR ZHURNAL VYSSHEI NERVNOI DEYATELNOSTI IMENI I P PAVLOVA) |
| **CU** “COUNTRY”  CU=( RUSSIA) |

**Table S6. Full description of the parameters of search related to the described schemes in Fig. S1. by Web of Science field tags (journals published in United States).**

| **Search parameters by Web of Science field tags** |
| --- |
|  |
| **WC** “Web of Science Category”  WC=(AGRICULTURE, DAIRY & ANIMAL SCIENCE OR AGRICULTURE, MULTIDISCIPLINARY OR BIOCHEMISTRY & MOLECULAR BIOLOGY OR BIOLOGY OR CHEMISTRY, MULTIDISCIPLINARY OR ENGINEERING, CHEMICAL OR GENETICS & HEREDITY OR MATHEMATICS OR MEDICINE, RESEARCH & EXPERIMENTAL OR MICROBIOLOGY OR NEUROSCIENCES OR PARASITOLOGY OR PHYSICS, MULTIDISCIPLINARY OR PSYCHIATRY OR PUBLIC, ENVIRONMENTAL & OCCUPATIONAL HEALTH OR SOCIAL SCIENCES, INTERDISCIPLINARY OR SOIL SCIENCE OR TROPICAL MEDICINE OR VETERINARY SCIENCES) |
| **SO** “Publication name”  SO=( AATCC REVIEW OR Abstract and Applied Analysis OR ABSTRACTS OF PAPERS OF THE AMERICAN CHEMICAL SOCIETY OR ACADEMIC PSYCHIATRY OR ACCOUNTS OF CHEMICAL RESEARCH OR ACS Chemical Biology OR ACS Chemical Neuroscience OR ACS Chemical Neuroscience OR ACS Combinatorial Science OR ACS Nano OR ACS Sustainable Chemistry & Engineering OR ACS Sustainable Chemistry & Engineering OR ACUPUNCTURE & ELECTRO-THERAPEUTICS RESEARCH OR Administration and Policy in Mental Health and Mental Health Services Research OR Adolescent Psychiatry OR ADVANCED NONLINEAR STUDIES OR Advances in Applied Microbiology OR Advances in Carbohydrate Chemistry and Biochemistry OR Advances in Difference Equations OR Advances in Differential Equations OR Advances in Experimental Medicine and Biology OR Advances in Experimental Medicine and Biology OR Advances in Genetics OR ADVANCES IN HUMAN GENETICS OR ADVANCES IN MATHEMATICS OR ADVANCES IN MICROBIAL ECOLOGY OR Advances in Microbial Physiology OR Advances in Microbial Physiology OR Advances in Parasitology OR ADVANCES IN POLYMER TECHNOLOGY OR Advances in Protein Chemistry and Structural Biology OR AEROSOL SCIENCE AND TECHNOLOGY OR AGRICULTURAL HISTORY OR Agroecology and Sustainable Food Systems OR AICHE JOURNAL OR AIDS AND BEHAVIOR OR AIDS EDUCATION AND PREVENTION OR AIDS PATIENT CARE AND STDS OR Algebra & Number Theory OR AMERICAN BEHAVIORAL SCIENTIST OR AMERICAN BIOLOGY TEACHER OR AMERICAN JOURNAL OF COMMUNITY PSYCHOLOGY OR AMERICAN JOURNAL OF EPIDEMIOLOGY OR AMERICAN JOURNAL OF EVALUATION OR AMERICAN JOURNAL OF GERIATRIC PSYCHIATRY OR AMERICAN JOURNAL OF GERIATRIC PSYCHIATRY OR AMERICAN JOURNAL OF HEALTH BEHAVIOR OR AMERICAN JOURNAL OF HEALTH PROMOTION OR AMERICAN JOURNAL OF HUMAN BIOLOGY OR AMERICAN JOURNAL OF HUMAN GENETICS OR AMERICAN JOURNAL OF INDUSTRIAL MEDICINE OR AMERICAN JOURNAL OF MATHEMATICS OR AMERICAN JOURNAL OF MEDICAL GENETICS PART A OR AMERICAN JOURNAL OF MEDICAL GENETICS PART B-NEUROPSYCHIATRIC GENETICS OR AMERICAN JOURNAL OF MEDICAL GENETICS PART B-NEUROPSYCHIATRIC GENETICS OR AMERICAN JOURNAL OF MEDICAL GENETICS PART C-SEMINARS IN MEDICAL GENETICS OR American Journal of Mens Health OR AMERICAN JOURNAL OF ORTHOPSYCHIATRY OR AMERICAN JOURNAL OF ORTHOPSYCHIATRY OR AMERICAN JOURNAL OF PHYSICS OR AMERICAN JOURNAL OF PREVENTIVE MEDICINE OR AMERICAN JOURNAL OF PSYCHIATRY OR AMERICAN JOURNAL OF PSYCHIATRY OR AMERICAN JOURNAL OF PUBLIC HEALTH OR AMERICAN JOURNAL OF PUBLIC HEALTH OR AMERICAN JOURNAL OF RESPIRATORY CELL AND MOLECULAR BIOLOGY OR American Journal of Translational Research OR AMERICAN JOURNAL OF TROPICAL MEDICINE AND HYGIENE OR AMERICAN JOURNAL OF TROPICAL MEDICINE AND HYGIENE OR AMERICAN JOURNAL OF VETERINARY RESEARCH OR AMERICAN MATHEMATICAL MONTHLY OR AMYLOID-JOURNAL OF PROTEIN FOLDING DISORDERS OR AMYLOID-JOURNAL OF PROTEIN FOLDING DISORDERS OR Analysis & PDE OR ANALYTICAL BIOCHEMISTRY OR ANIMAL BIOTECHNOLOGY OR ANNALS OF APPLIED BIOLOGY OR Annals of Clinical Psychiatry OR Annals of Clinical Psychiatry OR ANNALS OF EPIDEMIOLOGY OR Annals of Global Health OR ANNALS OF MATHEMATICS OR ANNALS OF NEUROLOGY OR ANNALS OF PHYSICS OR ANNALS OF THE AMERICAN ACADEMY OF POLITICAL AND SOCIAL SCIENCE OR Annual Review of Biochemistry OR Annual Review of Chemical and Biomolecular Engineering OR Annual Review of Genetics OR Annual Review of Genomics and Human Genetics OR Annual Review of Medicine OR Annual Review of Microbiology OR Annual Review of Neuroscience OR Annual Review of Public Health OR Annual Review of Public Health OR ANTIMICROBIAL AGENTS AND CHEMOTHERAPY OR ANTIOXIDANTS & REDOX SIGNALING OR APOPTOSIS OR APPLIED AND ENVIRONMENTAL MICROBIOLOGY OR APPLIED BIOCHEMISTRY AND BIOTECHNOLOGY OR ARCHAEA-AN INTERNATIONAL MICROBIOLOGICAL JOURNAL OR ARCHIVES OF BIOCHEMISTRY AND BIOPHYSICS OR Archives of Environmental & Occupational Health OR ARCHIVES OF INSECT BIOCHEMISTRY AND PHYSIOLOGY OR ARCHIVES OF MEDICAL RESEARCH OR ARCHIVES OF PATHOLOGY & LABORATORY MEDICINE OR ARCHIVES OF PSYCHIATRIC NURSING OR ARCHIVES OF PSYCHIATRIC NURSING OR ARCHIVES OF SEXUAL BEHAVIOR OR ARID LAND RESEARCH AND MANAGEMENT OR Asian Journal of Mathematics OR ASTROBIOLOGY OR ATOMIZATION AND SPRAYS OR AUDIOLOGY AND NEURO-OTOLOGY OR AVIAN DISEASES OR AVIATION SPACE AND ENVIRONMENTAL MEDICINE OR BEHAVIOR GENETICS OR BEHAVIORAL MEDICINE OR BEHAVIORAL MEDICINE OR BEHAVIORAL NEUROSCIENCE OR BEHAVIORAL NEUROSCIENCE OR Behavioral Sleep Medicine OR BEHAVIOURAL PHARMACOLOGY OR BEHAVIOURAL PHARMACOLOGY OR BIOCHEMICAL AND BIOPHYSICAL RESEARCH COMMUNICATIONS OR BIOCHEMICAL GENETICS OR BIOCHEMICAL GENETICS OR BIOCHEMISTRY OR BIOCHEMISTRY AND MOLECULAR BIOLOGY EDUCATION OR BIOCONJUGATE CHEMISTRY OR BIOCONJUGATE CHEMISTRY OR BIOELECTROMAGNETICS OR BIOESSAYS OR BIOESSAYS OR BIOFACTORS OR BIOLOGICAL BULLETIN OR BIOLOGICAL PSYCHIATRY OR BIOLOGICAL PSYCHIATRY OR BIOLOGICAL TRACE ELEMENT RESEARCH OR BIOLOGY BULLETIN OR BIOMACROMOLECULES OR Biomed Research International OR BIOMETRICS OR BIOORGANIC CHEMISTRY OR BIOPOLYMERS OR BIOSCIENCE OR BIOSECURITY AND BIOTERRORISM-BIODEFENSE STRATEGY PRACTICE AND SCIENCE OR BIOTECHNIQUES OR BIOTECHNOLOGY AND APPLIED BIOCHEMISTRY OR BIRTH DEFECTS RESEARCH PART B-DEVELOPMENTAL AND REPRODUCTIVE TOXICOLOGY OR Boundary Value Problems OR BRAIN AND COGNITION OR BRAIN AND LANGUAGE OR BRAIN BEHAVIOR AND IMMUNITY OR BRAIN RESEARCH BULLETIN OR Brain Stimulation OR BRAIN TOPOGRAPHY OR BULLETIN OF MATHEMATICAL BIOLOGY OR BULLETIN OF THE AMERICAN MATHEMATICAL SOCIETY OR BULLETIN OF THE MENNINGER CLINIC OR CALCULUS OF VARIATIONS AND PARTIAL DIFFERENTIAL EQUATIONS OR CALIFORNIA AGRICULTURE OR CANCER BIOTHERAPY AND RADIOPHARMACEUTICALS OR CANCER EPIDEMIOLOGY BIOMARKERS & PREVENTION OR Cancer Genetics OR CELL OR CELL BIOCHEMISTRY AND BIOPHYSICS OR CELL COMMUNICATION AND ADHESION OR Cell Host & Microbe OR Cell Host & Microbe OR CELL TRANSPLANTATION OR CELLULAR AND MOLECULAR NEUROBIOLOGY OR Cellular Reprogramming OR CEREBELLUM OR CEREBRAL CORTEX OR Channels OR CHEMICAL & ENGINEERING NEWS OR CHEMICAL & ENGINEERING NEWS OR CHEMICAL ENGINEERING OR CHEMICAL ENGINEERING COMMUNICATIONS OR CHEMICAL ENGINEERING PROGRESS OR CHEMICAL ENGINEERING SCIENCE OR CHEMICAL RESEARCH IN TOXICOLOGY OR CHEMICAL REVIEWS OR CHEMISTRY & BIOLOGY OR CHEMISTRY AND PHYSICS OF CARBON OR Chemosensory Perception OR CHILD AND ADOLESCENT PSYCHIATRIC CLINICS OF NORTH AMERICA OR CHILD PSYCHIATRY & HUMAN DEVELOPMENT OR CHILD PSYCHIATRY & HUMAN DEVELOPMENT OR CHILDRENS HEALTH CARE OR CHRONOBIOLOGY INTERNATIONAL OR Circulation-Cardiovascular Genetics OR CLAYS AND CLAY MINERALS OR Clinical and Vaccine Immunology OR CLINICAL AUTONOMIC RESEARCH OR Clinical Child Psychology and Psychiatry OR Clinical Child Psychology and Psychiatry OR CLINICAL DYSMORPHOLOGY OR CLINICAL EEG AND NEUROSCIENCE OR Clinical Gerontologist OR Clinical Gerontologist OR CLINICAL INFECTIOUS DISEASES OR CLINICAL MICROBIOLOGY REVIEWS OR Clothing and Textiles Research Journal OR CNS SPECTRUMS OR COGNITIVE AFFECTIVE & BEHAVIORAL NEUROSCIENCE OR Cognitive Computation OR Cold Spring Harbor Perspectives in Medicine OR COLOR RESEARCH AND APPLICATION OR COMBINATORICS PROBABILITY & COMPUTING OR COMBUSTION AND FLAME OR COMBUSTION SCIENCE AND TECHNOLOGY OR COMMUNICATIONS IN ALGEBRA OR COMMUNICATIONS IN ANALYSIS AND GEOMETRY OR Communications in Number Theory and Physics OR COMMUNICATIONS IN PARTIAL DIFFERENTIAL EQUATIONS OR COMMUNICATIONS IN SOIL SCIENCE AND PLANT ANALYSIS OR COMMUNICATIONS ON PURE AND APPLIED ANALYSIS OR COMMUNICATIONS ON PURE AND APPLIED MATHEMATICS OR COMPARATIVE BIOCHEMISTRY AND PHYSIOLOGY A-MOLECULAR & INTEGRATIVE PHYSIOLOGY OR COMPARATIVE BIOCHEMISTRY AND PHYSIOLOGY B-BIOCHEMISTRY & MOLECULAR BIOLOGY OR COMPARATIVE BIOCHEMISTRY AND PHYSIOLOGY C-TOXICOLOGY & PHARMACOLOGY OR Comparative Biochemistry and Physiology D-Genomics & Proteomics OR Comparative Biochemistry and Physiology D-Genomics & Proteomics OR COMPARATIVE MEDICINE OR COMPARATIVE PARASITOLOGY OR Compendium-Continuing Education for Veterinarians OR COMPOST SCIENCE & UTILIZATION OR COMPREHENSIVE PSYCHIATRY OR COMPREHENSIVE PSYCHIATRY OR Computational Intelligence and Neuroscience OR COMPUTERS & CHEMICAL ENGINEERING OR COMPUTERS IN BIOLOGY AND MEDICINE OR CONSTRUCTIVE APPROXIMATION OR Contemporary Clinical Trials OR CONTEMPORARY PSYCHOANALYSIS OR CONTINUITY AND CHANGE OR CRITICAL REVIEW OR CRITICAL REVIEWS IN BIOCHEMISTRY AND MOLECULAR BIOLOGY OR CRITICAL REVIEWS IN EUKARYOTIC GENE EXPRESSION OR CRITICAL REVIEWS IN MICROBIOLOGY OR CROSS-CULTURAL RESEARCH OR CRYOBIOLOGY OR CRYSTAL GROWTH & DESIGN OR CTS-Clinical and Translational Science OR CURRENT BIOLOGY OR CURRENT GENETICS OR CURRENT MICROBIOLOGY OR Current Neurology and Neuroscience Reports OR CURRENT OPINION IN GENETICS & DEVELOPMENT OR CURRENT OPINION IN NEUROLOGY OR CURRENT OPINION IN PSYCHIATRY OR CURRENT OPINION IN PSYCHIATRY OR Current Psychiatry Reports OR Current Psychiatry Reports OR Current Topics in Membranes OR Current Topics in Microbiology and Immunology OR DAEDALUS OR DEPRESSION AND ANXIETY OR DEPRESSION AND ANXIETY OR Developmental Disabilities Research Reviews OR Developmental Disabilities Research Reviews OR Developmental Neurobiology OR DIABETES EDUCATOR OR DIAGNOSTIC MICROBIOLOGY AND INFECTIOUS DISEASE OR DIAGNOSTIC MOLECULAR PATHOLOGY OR Differential and Integral Equations OR Disability and Health Journal OR Disability and Health Journal OR Disaster Medicine and Public Health Preparedness OR Disaster Medicine and Public Health Preparedness OR Disaster Prevention and Management OR Discovery Medicine OR DISCRETE & COMPUTATIONAL GEOMETRY OR DISCRETE AND CONTINUOUS DYNAMICAL SYSTEMS OR DNA AND CELL BIOLOGY OR DNA AND CELL BIOLOGY OR DOKLADY CHEMISTRY OR DOMESTIC ANIMAL ENDOCRINOLOGY OR DRUG AND CHEMICAL TOXICOLOGY OR DRYING TECHNOLOGY OR DUKE MATHEMATICAL JOURNAL OR DYNAMIC SYSTEMS AND APPLICATIONS OR Eating Disorders OR Eating Disorders OR ELECTROMAGNETIC BIOLOGY AND MEDICINE OR ELECTRONIC JOURNAL OF COMBINATORICS OR Electronic Journal of Differential Equations OR Electronic Journal of Linear Algebra OR Electronic Research Announcements in Mathematical Sciences OR EMBO JOURNAL OR ENERGY & FUELS OR Energy Sources Part A-Recovery Utilization and Environmental Effects OR ENVIRONMENTAL AND MOLECULAR MUTAGENESIS OR ENVIRONMENTAL HEALTH PERSPECTIVES OR Environmental Progress & Sustainable Energy OR ENVIRONMENTAL RESEARCH OR EPIDEMIOLOGIC REVIEWS OR EPIDEMIOLOGY OR EPIDEMIOLOGY OR Epigenetics OR Epigenetics OR EPILEPSY & BEHAVIOR OR ETHNICITY & DISEASE OR EUKARYOTIC CELL OR Evaluation OR EVALUATION AND PROGRAM PLANNING OR EVALUATION REVIEW OR EVOLUTION OR EVOLUTION & DEVELOPMENT OR EVOLUTIONARY ECOLOGY RESEARCH OR EXPERIMENTAL AND CLINICAL PSYCHOPHARMACOLOGY OR EXPERIMENTAL HEMATOLOGY OR EXPERIMENTAL MATHEMATICS OR EXPERIMENTAL NEUROLOGY OR EXPERIMENTAL PARASITOLOGY OR Families Systems & Health OR Families Systems & Health OR FAMILY & COMMUNITY HEALTH OR FASEB JOURNAL OR FASEB JOURNAL OR Field Methods OR FINITE FIELDS AND THEIR APPLICATIONS OR Fixed Point Theory and Applications OR FLY OR Food and Environmental Virology OR FOUNDATIONS OF COMPUTATIONAL MATHEMATICS OR FOUNDATIONS OF PHYSICS OR FREE RADICAL BIOLOGY AND MEDICINE OR Frontiers in Bioscience-Landmark OR FRONTIERS IN NEUROENDOCRINOLOGY OR FUNGAL GENETICS AND BIOLOGY OR FUTURE OF CHILDREN OR G3-Genes Genomes Genetics OR GENE EXPRESSION OR GENERAL HOSPITAL PSYCHIATRY OR GENERAL HOSPITAL PSYCHIATRY OR GENERAL RELATIVITY AND GRAVITATION OR GENES & DEVELOPMENT OR Genes and Nutrition OR GENES CHROMOSOMES & CANCER OR GENESIS OR GENETIC EPIDEMIOLOGY OR GENETIC EPIDEMIOLOGY OR Genetic Testing and Molecular Biomarkers OR GENETICS OR GENETICS IN MEDICINE OR GENOME RESEARCH OR GENOME RESEARCH OR GENOMICS OR GEOMETRY & TOPOLOGY OR GLIA OR Global Health Promotion OR GLQ-A JOURNAL OF LESBIAN AND GAY STUDIES OR GLYCOBIOLOGY OR HARVARD REVIEW OF PSYCHIATRY OR HARVARD REVIEW OF PSYCHIATRY OR HEALTH & PLACE OR HEALTH & PLACE OR Health and Human Rights OR Health Care for Women International OR HEALTH EDUCATION & BEHAVIOR OR HEALTH PHYSICS OR HEALTH PHYSICS OR HEMOGLOBIN OR HERD-Health Environments Research & Design Journal OR HETEROATOM CHEMISTRY OR HIGH ALTITUDE MEDICINE & BIOLOGY OR HIPPOCAMPUS OR HISTORIA MATHEMATICA OR Homology Homotopy and Applications OR HOUSTON JOURNAL OF MATHEMATICS OR HUMAN BIOLOGY OR HUMAN BIOLOGY OR HUMAN BRAIN MAPPING OR HUMAN GENE THERAPY OR HUMAN GENE THERAPY OR Human Gene Therapy Clinical Development OR Human Gene Therapy Methods OR Human Gene Therapy Methods OR HUMAN GENETICS OR HUMAN MUTATION OR HUMAN ORGANIZATION OR IEEE Transactions on Autonomous Mental Development OR ILLINOIS JOURNAL OF MATHEMATICS OR INDIANA UNIVERSITY MATHEMATICS JOURNAL OR INDUSTRIAL & ENGINEERING CHEMISTRY RESEARCH OR INFECTION CONTROL AND HOSPITAL EPIDEMIOLOGY OR Integrative Psychological and Behavioral Science OR INTERNATIONAL CLINICAL PSYCHOPHARMACOLOGY OR INTERNATIONAL JOURNAL OF ALGEBRA AND COMPUTATION OR International Journal of Applied Research in Veterinary Medicine OR International Journal of Astrobiology OR International Journal of Chemical Reactor Engineering OR International Journal of Clinical and Experimental Medicine OR International Journal of Cognitive Therapy OR INTERNATIONAL JOURNAL OF EATING DISORDERS OR INTERNATIONAL JOURNAL OF EATING DISORDERS OR International Journal of Genomics OR International Journal of Genomics OR INTERNATIONAL JOURNAL OF INTERCULTURAL RELATIONS OR International Journal of Mental Health and Addiction OR International Journal of Mental Health and Addiction OR INTERNATIONAL JOURNAL OF MOLECULAR MEDICINE OR INTERNATIONAL JOURNAL OF OCCUPATIONAL AND ENVIRONMENTAL HEALTH OR INTERNATIONAL JOURNAL OF OCCUPATIONAL AND ENVIRONMENTAL HEALTH OR International Journal of Peptide Research and Therapeutics OR INTERNATIONAL JOURNAL OF PSYCHIATRY IN MEDICINE OR INTERNATIONAL JOURNAL OF PSYCHIATRY IN MEDICINE OR International Journal of Sexual Health OR International Journal of Sexual Health OR INTERNATIONAL JOURNAL OF THEORETICAL PHYSICS OR International Perspectives on Sexual and Reproductive Health OR INTERNATIONAL PSYCHOGERIATRICS OR International Review of Cell and Molecular Biology OR International Review of Neurobiology OR IUBMB LIFE OR JAMA Psychiatry OR JAMA Psychiatry OR JARO-JOURNAL OF THE ASSOCIATION FOR RESEARCH IN OTOLARYNGOLOGY OR JAVMA-JOURNAL OF THE AMERICAN VETERINARY MEDICAL ASSOCIATION OR JOURNAL OF ADOLESCENT HEALTH OR JOURNAL OF ADOLESCENT HEALTH OR JOURNAL OF AGRICULTURAL AND FOOD CHEMISTRY OR JOURNAL OF AGRICULTURAL BIOLOGICAL AND ENVIRONMENTAL STATISTICS OR Journal of Agromedicine OR JOURNAL OF ALGEBRA OR JOURNAL OF ALGEBRAIC COMBINATORICS OR JOURNAL OF ALGEBRAIC GEOMETRY OR JOURNAL OF AMERICAN COLLEGE HEALTH OR JOURNAL OF ANIMAL SCIENCE OR JOURNAL OF ANXIETY DISORDERS OR JOURNAL OF APPLIED ANIMAL WELFARE SCIENCE OR JOURNAL OF APPLIED POULTRY RESEARCH OR JOURNAL OF APPROXIMATION THEORY OR JOURNAL OF AQUATIC ANIMAL HEALTH OR JOURNAL OF ASSISTED REPRODUCTION AND GENETICS OR Journal of Attention Disorders OR Journal of Attention Disorders OR JOURNAL OF AVIAN MEDICINE AND SURGERY OR JOURNAL OF BACTERIOLOGY OR JOURNAL OF BEHAVIORAL HEALTH SERVICES & RESEARCH OR JOURNAL OF BEHAVIORAL HEALTH SERVICES & RESEARCH OR JOURNAL OF BIOCHEMICAL AND MOLECULAR TOXICOLOGY OR JOURNAL OF BIOLOGICAL CHEMISTRY OR JOURNAL OF BIOLOGICAL EDUCATION OR JOURNAL OF BIOMOLECULAR STRUCTURE & DYNAMICS OR JOURNAL OF BLACK STUDIES OR JOURNAL OF CANCER EDUCATION OR JOURNAL OF CARBOHYDRATE CHEMISTRY OR Journal of Cardiovascular Translational Research OR JOURNAL OF CATALYSIS OR JOURNAL OF CELLULAR BIOCHEMISTRY OR JOURNAL OF CEREBRAL BLOOD FLOW AND METABOLISM OR JOURNAL OF CHEMICAL AND ENGINEERING DATA OR JOURNAL OF CHEMICAL AND ENGINEERING DATA OR JOURNAL OF CHEMICAL EDUCATION OR Journal of Chemical Information and Modeling OR Journal of Chemistry OR JOURNAL OF CHILD AND ADOLESCENT PSYCHOPHARMACOLOGY OR Journal of Child and Family Studies OR JOURNAL OF CLINICAL EPIDEMIOLOGY OR JOURNAL OF CLINICAL INVESTIGATION OR JOURNAL OF CLINICAL MICROBIOLOGY OR JOURNAL OF CLINICAL NEUROPHYSIOLOGY OR JOURNAL OF CLINICAL PSYCHIATRY OR JOURNAL OF CLINICAL PSYCHIATRY OR JOURNAL OF CLINICAL PSYCHOPHARMACOLOGY OR JOURNAL OF COGNITIVE NEUROSCIENCE OR JOURNAL OF COMBINATORIAL DESIGNS OR JOURNAL OF COMBINATORIAL THEORY SERIES A OR JOURNAL OF COMBINATORIAL THEORY SERIES B OR JOURNAL OF COMMUNITY HEALTH OR JOURNAL OF COMMUNITY PSYCHOLOGY OR Journal of Commutative Algebra OR JOURNAL OF COMPARATIVE NEUROLOGY OR Journal of Computational and Theoretical Nanoscience OR JOURNAL OF COMPUTATIONAL CHEMISTRY OR JOURNAL OF COMPUTATIONAL NEUROSCIENCE OR JOURNAL OF CONSCIOUSNESS STUDIES OR Journal of Correctional Health Care OR JOURNAL OF DAIRY SCIENCE OR Journal of Diabetes Research OR JOURNAL OF DIFFERENTIAL EQUATIONS OR JOURNAL OF DIFFERENTIAL GEOMETRY OR Journal of Dual Diagnosis OR Journal of Dynamics and Differential Equations OR Journal of East Asian Studies OR JOURNAL OF ECT OR JOURNAL OF ECT OR Journal of Energetic Materials OR JOURNAL OF ENVIRONMENTAL HEALTH OR JOURNAL OF ENVIRONMENTAL SCIENCE AND HEALTH PART B-PESTICIDES FOOD CONTAMINANTS AND AGRICULTURAL WASTES OR JOURNAL OF EQUINE VETERINARY SCIENCE OR JOURNAL OF ETHNOBIOLOGY OR JOURNAL OF EUKARYOTIC MICROBIOLOGY OR Journal of Exotic Pet Medicine OR JOURNAL OF EXPERIMENTAL MEDICINE OR Journal of Exposure Science and Environmental Epidemiology OR JOURNAL OF FOOD BIOCHEMISTRY OR JOURNAL OF FOOD PROCESS ENGINEERING OR Journal of Function Spaces OR JOURNAL OF FUNCTIONAL ANALYSIS OR Journal of Genetic Counseling OR JOURNAL OF GEOMETRIC ANALYSIS OR JOURNAL OF GERIATRIC PSYCHIATRY AND NEUROLOGY OR JOURNAL OF GRAPH THEORY OR JOURNAL OF HEALTH AND SOCIAL BEHAVIOR OR JOURNAL OF HEALTH CARE FOR THE POOR AND UNDERSERVED OR JOURNAL OF HEREDITY OR JOURNAL OF HOMOSEXUALITY OR Journal of Immigrant and Minority Health OR JOURNAL OF IMMUNOTHERAPY OR JOURNAL OF INEQUALITIES AND APPLICATIONS OR JOURNAL OF INFECTIOUS DISEASES OR JOURNAL OF INORGANIC BIOCHEMISTRY OR Journal of Integral Equations and Applications OR JOURNAL OF INTERFERON AND CYTOKINE RESEARCH OR JOURNAL OF INVESTIGATIVE MEDICINE OR JOURNAL OF LIPID RESEARCH OR JOURNAL OF LIPOSOME RESEARCH OR JOURNAL OF MATHEMATICAL ANALYSIS AND APPLICATIONS OR JOURNAL OF MATHEMATICAL CHEMISTRY OR JOURNAL OF MEDICAL ENTOMOLOGY OR JOURNAL OF MEMBRANE BIOLOGY OR JOURNAL OF MICROWAVE POWER AND ELECTROMAGNETIC ENERGY OR Journal of Mixed Methods Research OR Journal of Modern Dynamics OR JOURNAL OF MOLECULAR BIOLOGY OR JOURNAL OF MOLECULAR GRAPHICS & MODELLING OR JOURNAL OF MOLECULAR NEUROSCIENCE OR JOURNAL OF MOLECULAR NEUROSCIENCE OR JOURNAL OF MOTOR BEHAVIOR OR JOURNAL OF NANOSCIENCE AND NANOTECHNOLOGY OR JOURNAL OF NERVOUS AND MENTAL DISEASE OR JOURNAL OF NERVOUS AND MENTAL DISEASE OR Journal of Neurodevelopmental Disorders OR Journal of Neuroimmune Pharmacology OR JOURNAL OF NEUROLINGUISTICS OR JOURNAL OF NEUROPATHOLOGY AND EXPERIMENTAL NEUROLOGY OR JOURNAL OF NEUROPHYSIOLOGY OR JOURNAL OF NEUROPHYSIOLOGY OR JOURNAL OF NEUROPSYCHIATRY AND CLINICAL NEUROSCIENCES OR JOURNAL OF NEUROPSYCHIATRY AND CLINICAL NEUROSCIENCES OR JOURNAL OF NEUROSCIENCE OR JOURNAL OF NEUROSCIENCE OR JOURNAL OF NEUROSCIENCE RESEARCH OR JOURNAL OF NEUROTRAUMA OR JOURNAL OF NEUROVIROLOGY OR JOURNAL OF NUMBER THEORY OR JOURNAL OF NUTRITIONAL BIOCHEMISTRY OR JOURNAL OF OCCUPATIONAL AND ENVIRONMENTAL HYGIENE OR JOURNAL OF OCCUPATIONAL AND ENVIRONMENTAL MEDICINE OR Journal of Occupational Health Psychology OR Journal of Ophthalmology OR JOURNAL OF PAIN OR JOURNAL OF PARASITOLOGY OR JOURNAL OF PERSONALITY DISORDERS OR JOURNAL OF PHARMACEUTICAL SCIENCES OR Journal of Physical Activity & Health OR JOURNAL OF PHYSICAL AND CHEMICAL REFERENCE DATA OR JOURNAL OF PHYSICAL AND CHEMICAL REFERENCE DATA OR JOURNAL OF PHYSICS AND CHEMISTRY OF SOLIDS OR Journal of Primary Prevention OR Journal of Psychiatric Practice OR JOURNAL OF PUBLIC HEALTH DENTISTRY OR JOURNAL OF PUBLIC HEALTH MANAGEMENT AND PRACTICE OR JOURNAL OF RECEPTORS AND SIGNAL TRANSDUCTION OR JOURNAL OF RELIGION & HEALTH OR JOURNAL OF RURAL HEALTH OR JOURNAL OF RURAL HEALTH OR JOURNAL OF SAFETY RESEARCH OR JOURNAL OF SAFETY RESEARCH OR JOURNAL OF SCHOOL HEALTH OR JOURNAL OF SCHOOL HEALTH OR JOURNAL OF SEX RESEARCH OR JOURNAL OF SOIL AND WATER CONSERVATION OR Journal of Stroke & Cerebrovascular Diseases OR JOURNAL OF STRUCTURAL BIOLOGY OR JOURNAL OF SURFACTANTS AND DETERGENTS OR JOURNAL OF SWINE HEALTH AND PRODUCTION OR Journal of Symplectic Geometry OR JOURNAL OF THE AMERICAN ACADEMY OF PSYCHIATRY AND THE LAW OR JOURNAL OF THE AMERICAN ANIMAL HOSPITAL ASSOCIATION OR Journal of the American Association for Laboratory Animal Science OR JOURNAL OF THE AMERICAN CHEMICAL SOCIETY OR JOURNAL OF THE AMERICAN MATHEMATICAL SOCIETY OR Journal of the American Psychiatric Nurses Association OR Journal of the American Psychiatric Nurses Association OR JOURNAL OF THE AMERICAN PSYCHOANALYTIC ASSOCIATION OR Journal of the History of the Neurosciences OR JOURNAL OF THE INTERNATIONAL NEUROPSYCHOLOGICAL SOCIETY OR JOURNAL OF THE INTERNATIONAL NEUROPSYCHOLOGICAL SOCIETY OR JOURNAL OF THE PAKISTAN MEDICAL ASSOCIATION OR JOURNAL OF THE PERIPHERAL NERVOUS SYSTEM OR JOURNAL OF THEORETICAL BIOLOGY OR JOURNAL OF TOXICOLOGY AND ENVIRONMENTAL HEALTH-PART A-CURRENT ISSUES OR JOURNAL OF TOXICOLOGY AND ENVIRONMENTAL HEALTH-PART B-CRITICAL REVIEWS OR Journal of Trauma & Dissociation OR JOURNAL OF TRAUMATIC STRESS OR JOURNAL OF URBAN HEALTH-BULLETIN OF THE NEW YORK ACADEMY OF MEDICINE OR JOURNAL OF VESTIBULAR RESEARCH-EQUILIBRIUM & ORIENTATION OR JOURNAL OF VETERINARY DENTISTRY OR JOURNAL OF VETERINARY DIAGNOSTIC INVESTIGATION OR JOURNAL OF VETERINARY EMERGENCY AND CRITICAL CARE OR JOURNAL OF VETERINARY INTERNAL MEDICINE OR JOURNAL OF VETERINARY MEDICAL EDUCATION OR JOURNAL OF WILDLIFE DISEASES OR JOURNAL OF WOMENS HEALTH OR JOURNAL OF WOMENS HEALTH OR JOURNAL OF ZOO AND WILDLIFE MEDICINE OR Kinetic and Related Models OR LAB ANIMAL OR LABORATORY INVESTIGATION OR LANGMUIR OR LARYNGOSCOPE OR LEARNING & MEMORY OR Lecture Notes in Mathematics OR Lymphatic Research and Biology OR mAbs OR MALARIA JOURNAL OR MALARIA JOURNAL OR MAMMALIAN GENOME OR MAMMALIAN GENOME OR MATERNAL AND CHILD HEALTH JOURNAL OR MATHEMATICAL BIOSCIENCES OR Mathematical Control and Related Fields OR MATHEMATICAL INTELLIGENCER OR MATHEMATICAL RESEARCH LETTERS OR mBio OR MEDICAL ANTHROPOLOGY QUARTERLY OR MEDICAL CARE OR MEDICAL SCIENCE MONITOR OR MEDICC Review OR MEDICC Review OR MELANOMA RESEARCH OR MEMOIRS OF THE AMERICAN MATHEMATICAL SOCIETY OR METABOLIC BRAIN DISEASE OR Metabolic Syndrome and Related Disorders OR METHODS OR Methods in Enzymology OR MICHIGAN MATHEMATICAL JOURNAL OR Microbial Biotechnology OR Microbial Drug Resistance OR MICROBIAL ECOLOGY OR MICROBIOLOGY AND MOLECULAR BIOLOGY REVIEWS OR MICROSCOPY RESEARCH AND TECHNIQUE OR MMWR Recommendations and Reports OR MMWR Surveillance Summaries OR MMWR-MORBIDITY AND MORTALITY WEEKLY REPORT OR MOLECULAR AND CELLULAR BIOLOGY OR MOLECULAR AND CELLULAR NEUROSCIENCE OR MOLECULAR BIOLOGY AND EVOLUTION OR MOLECULAR BIOLOGY AND EVOLUTION OR MOLECULAR BIOTECHNOLOGY OR MOLECULAR CARCINOGENESIS OR MOLECULAR CELL OR MOLECULAR GENETICS AND METABOLISM OR MOLECULAR GENETICS AND METABOLISM OR MOLECULAR GENETICS AND METABOLISM OR Molecular Genetics Microbiology and Virology OR Molecular Genetics Microbiology and Virology OR MOLECULAR MEDICINE OR MOLECULAR MEDICINE OR MOLECULAR MEMBRANE BIOLOGY OR MOLECULAR NEUROBIOLOGY OR MOLECULAR PHARMACEUTICS OR MOLECULAR PHYLOGENETICS AND EVOLUTION OR MOLECULAR PHYLOGENETICS AND EVOLUTION OR MOLECULAR PLANT-MICROBE INTERACTIONS OR MOLECULAR REPRODUCTION AND DEVELOPMENT OR Molecular Systems Biology OR MOLECULAR THERAPY OR MOLECULAR THERAPY OR Molecular Therapy-Nucleic Acids OR MOLECULAR VISION OR MOTOR CONTROL OR MUSCLE & NERVE OR NANO LETTERS OR Nature Chemical Biology OR NATURE GENETICS OR NATURE MEDICINE OR NATURE MEDICINE OR NATURE NEUROSCIENCE OR NATURE STRUCTURAL & MOLECULAR BIOLOGY OR NEGOTIATION JOURNAL OR NEURAL COMPUTATION OR NEURAL PLASTICITY OR NEUROBIOLOGY OF LEARNING AND MEMORY OR NEUROCASE OR NEUROCHEMICAL RESEARCH OR NEUROCHEMICAL RESEARCH OR NEUROIMAGE OR NEUROIMAGING CLINICS OF NORTH AMERICA OR NEUROINFORMATICS OR NEUROLOGIC CLINICS OR NEUROLOGICAL RESEARCH OR NEUROMODULATION OR NEUROMOLECULAR MEDICINE OR NEUROMUSCULAR DISORDERS OR NEURON OR NEUROPSYCHOLOGY OR NEUROPSYCHOLOGY REVIEW OR NEUROREPORT OR NEUROSCIENTIST OR Neurotherapeutics OR NEUROTOXICITY RESEARCH OR New York Journal of Mathematics OR NITRIC OXIDE-BIOLOGY AND CHEMISTRY OR Notre Dame Journal of Formal Logic OR Nucleic Acid Therapeutics OR Nucleic Acid Therapeutics OR NUCLEOSIDES NUCLEOTIDES & NUCLEIC ACIDS OR OMICS-A JOURNAL OF INTEGRATIVE BIOLOGY OR OPHTHALMIC GENETICS OR Organogenesis OR PACIFIC JOURNAL OF MATHEMATICS OR PARTICULATE SCIENCE AND TECHNOLOGY OR PEPTIDES OR PERSPECTIVES IN BIOLOGY AND MEDICINE OR PERSPECTIVES IN PSYCHIATRIC CARE OR PERSPECTIVES IN PSYCHIATRIC CARE OR PESTICIDE BIOCHEMISTRY AND PHYSIOLOGY OR PETROLEUM SCIENCE AND TECHNOLOGY OR Pharmacogenetics and Genomics OR PHARMACOGENOMICS JOURNAL OR PHOTOCHEMISTRY AND PHOTOBIOLOGY OR PHYSICAL REVIEW LETTERS OR Physical Review X OR PHYSICS TODAY OR PHYSIOLOGICAL GENOMICS OR PLANT CELL OR Plant Genome OR PLASMA CHEMISTRY AND PLASMA PROCESSING OR PLASMID OR PLOS BIOLOGY OR PLOS BIOLOGY OR PLoS Genetics OR PLoS Neglected Tropical Diseases OR PLoS Neglected Tropical Diseases OR PLoS Pathogens OR PLoS Pathogens OR POLYMER ENGINEERING AND SCIENCE OR POULTRY SCIENCE OR PPAR Research OR Prehospital Emergency Care OR PREPARATIVE BIOCHEMISTRY & BIOTECHNOLOGY OR Preventing Chronic Disease OR Preventing Chronic Disease OR PREVENTION SCIENCE OR PREVENTIVE MEDICINE OR Prion OR PROCEEDINGS OF THE AMERICAN MATHEMATICAL SOCIETY OR PROCEEDINGS OF THE BIOLOGICAL SOCIETY OF WASHINGTON OR PROCEEDINGS OF THE COMBUSTION INSTITUTE OR PROCESS SAFETY PROGRESS OR Progress in Community Health Partnerships-Research Education and Action OR Progress in Molecular Biology and Translational Science OR PROSTAGLANDINS & OTHER LIPID MEDIATORS OR PROTEIN EXPRESSION AND PURIFICATION OR PROTEIN JOURNAL OR PROTEIN SCIENCE OR PROTEINS-STRUCTURE FUNCTION AND BIOINFORMATICS OR PSYCHIATRIC ANNALS OR PSYCHIATRIC CLINICS OF NORTH AMERICA OR PSYCHIATRIC GENETICS OR PSYCHIATRIC GENETICS OR PSYCHIATRIC QUARTERLY OR PSYCHIATRIC REHABILITATION JOURNAL OR PSYCHIATRIC SERVICES OR PSYCHIATRIC SERVICES OR PSYCHIATRIC SERVICES OR PSYCHIATRY-INTERPERSONAL AND BIOLOGICAL PROCESSES OR PSYCHIATRY-INTERPERSONAL AND BIOLOGICAL PROCESSES OR PSYCHOLOGICAL MEDICINE OR PSYCHOLOGICAL MEDICINE OR Psychological Trauma-Theory Research Practice and Policy OR PSYCHOPHARMACOLOGY BULLETIN OR PSYCHOPHARMACOLOGY BULLETIN OR PSYCHOPHYSIOLOGY OR PSYCHOSOMATIC MEDICINE OR PSYCHOSOMATIC MEDICINE OR PSYCHOSOMATICS OR PSYCHOSOMATICS OR PUBLIC HEALTH NURSING OR PUBLIC HEALTH NURSING OR PUBLIC HEALTH REPORTS OR PUBLIC HEALTH REPORTS OR Puerto Rico Health Sciences Journal OR Pure and Applied Mathematics Quarterly OR QUALITATIVE INQUIRY OR Qualitative Research OR Quantum Information Processing OR QUARTERLY REVIEW OF BIOLOGY OR RADIATION RESEARCH OR RANDOM STRUCTURES & ALGORITHMS OR Representation Theory OR Research in Social & Administrative Pharmacy OR Review of Symbolic Logic OR REVIEWS IN COMPUTATIONAL CHEMISTRY OR REVIEWS OF MODERN PHYSICS OR REVISTA PANAMERICANA DE SALUD PUBLICA-PAN AMERICAN JOURNAL OF PUBLIC HEALTH OR RISK ANALYSIS OR RNA Biology OR RNA-A PUBLICATION OF THE RNA SOCIETY OR ROCKY MOUNTAIN JOURNAL OF MATHEMATICS OR SCHIZOPHRENIA BULLETIN OR SCHIZOPHRENIA BULLETIN OR SCIENCE & SOCIETY OR Science Signaling OR Science Translational Medicine OR SEPARATION AND PURIFICATION REVIEWS OR SEPARATION SCIENCE AND TECHNOLOGY OR SEPARATION SCIENCE AND TECHNOLOGY OR Sexuality Research and Social Policy OR SLEEP OR SOCIAL RESEARCH OR SOCIAL SCIENCE COMPUTER REVIEW OR Social Work in Public Health OR SOCIETY OR SOIL SCIENCE OR SOIL SCIENCE SOCIETY OF AMERICA JOURNAL OR SOLVENT EXTRACTION AND ION EXCHANGE OR Standards in Genomic Sciences OR Standards in Genomic Sciences OR Statistical Applications in Genetics and Molecular Biology OR Stem Cell Reviews and Reports OR STEM CELLS AND DEVELOPMENT OR STEROIDS OR STRUCTURAL CHEMISTRY OR STUDIES IN FAMILY PLANNING OR SUBSTANCE USE & MISUSE OR SUICIDE AND LIFE-THREATENING BEHAVIOR OR SYNAPSE OR THEORETICAL POPULATION BIOLOGY OR THERIOGENOLOGY OR Topics in Companion Animal Medicine OR Topics in Current Chemistry OR TOXICOLOGY AND INDUSTRIAL HEALTH OR Traffic Injury Prevention OR Traffic Injury Prevention OR TRANSACTIONS OF THE AMERICAN MATHEMATICAL SOCIETY OR TRANSFORMATION GROUPS OR Translational Psychiatry OR Translational Research OR Translational Stroke Research OR TRENDS IN MICROBIOLOGY OR TRENDS IN MICROBIOLOGY OR TRENDS IN PARASITOLOGY OR TRIBOLOGY LETTERS OR Undersea and Hyperbaric Medicine OR VADOSE ZONE JOURNAL OR VECTOR-BORNE AND ZOONOTIC DISEASES OR VETERINARY CLINICAL PATHOLOGY OR VETERINARY CLINICS OF NORTH AMERICA-EQUINE PRACTICE OR VETERINARY CLINICS OF NORTH AMERICA-FOOD ANIMAL PRACTICE OR VETERINARY CLINICS OF NORTH AMERICA-SMALL ANIMAL PRACTICE OR VETERINARY OPHTHALMOLOGY OR VETERINARY PATHOLOGY OR VETERINARY RADIOLOGY & ULTRASOUND OR VETERINARY SURGERY OR Virulence OR VIRUS GENES OR VISUAL NEUROSCIENCE OR Vitamins and Hormones OR WAVE MOTION OR WILDERNESS & ENVIRONMENTAL MEDICINE OR Wiley Interdisciplinary Reviews-Nanomedicine and Nanobiotechnology OR Wiley Interdisciplinary Reviews-Systems Biology and Medicine OR WOMEN & HEALTH OR WOMENS HEALTH ISSUES OR WOUND REPAIR AND REGENERATION OR YOUTH & SOCIETY OR ZOO BIOLOGY) |
| **CU** “COUNTRY”  CU=( USA) |
