# Supplementary material for: Brazilian Science between National and Foreign Journals: Methodology for Analyzing the Production and Impact in Emerging Scientific Communities
Source: PLoS One. 2016 May 12;11(5):e0155148. doi: 10.1371/journal.pone.0155148 (PMC4865143; doi:10.1371/journal.pone.0155148)
Supplement: S2 Appendix — (DOCX) [file pone.0155148.s002.docx]

**S2 Appendix: Layout and descriptions of the search strategies and analysis resources of the Web of Science utilized for the composition of Sample 2.**

Fig. S1 presents the layout of the search strategy and describes how we utilize the analysis resources of the Web of Science for the Sample 2 composition. Table S1 complements the Fig. S1 with a full description of the search parameters by Web of Science field tags.

The Sample 2 data were collected from WoS on 01-13-2014, totalizing 85,082 papers. However, as mentioned above, considering that WoS is updated almost every working day, the obtained figures will constantly depend on the day in which the database was accessed. Thus, when we collected the data on 13^rd^ April, 2016 we obtained 94,767 papers observing the criteria for compose the Sample 1. As commented above, one could argue that the difference between these results is probably due to the increased number of papers indexed and covered by WoS . It is important to emphasize that this reduction of the sample size does not change the conclusions drawn from our analysis.


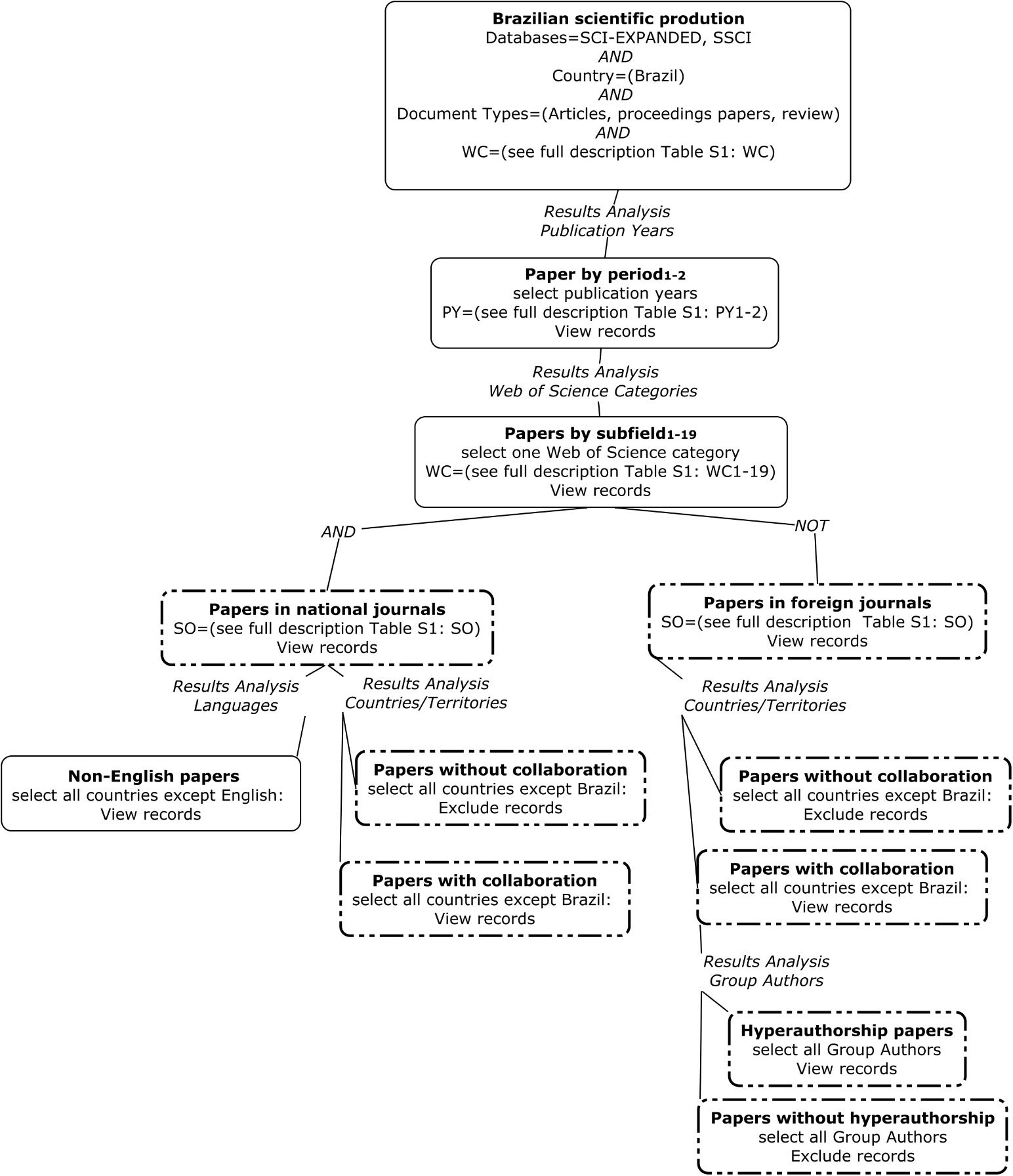


**Fig. S1. Strategy layout of search and analysis resources utilized by the Brazilian papers sample composition according to national or foreign publication, language, international collaboration and author-groups participation**

The dashed borders represent the data which generate “Citation reports” to select impact information according to the described formula in the section “Materials and Methods” of the paper.

**Table S1. Full description of the search parameters related to the described layout in Fig. S1 by Web of Science field tags.**

| **Search parameters by Web of Science field tags** |
| --- |
|  |
| **WC** “Web of Science Category”  WC=(AGRICULTURE, DAIRY & ANIMAL SCIENCE OR AGRICULTURE, MULTIDISCIPLINARY OR BIOCHEMISTRY & MOLECULAR BIOLOGY OR BIOLOGY OR CHEMISTRY, MULTIDISCIPLINARY OR ENGINEERING, CHEMICAL OR GENETICS & HEREDITY OR MATHEMATICS OR MEDICINE, RESEARCH & EXPERIMENTAL OR MICROBIOLOGY OR NEUROSCIENCES OR PARASITOLOGY OR PHYSICS, MULTIDISCIPLINARY OR PSYCHIATRY OR PUBLIC, ENVIRONMENTAL & OCCUPATIONAL HEALTH OR SOCIAL SCIENCES, INTERDISCIPLINARY OR SOIL SCIENCE OR TROPICAL MEDICINE OR VETERINARY SCIENCES) |
| **PY** “Publication Years”  PY_1_=2002-2006  PY_2_=2007-2011 |
| **WC** “Web of Science Category”  WC_1_= AGRICULTURE, DAIRY & ANIMAL SCIENCE  WC_2_= AGRICULTURE, MULTIDISCIPLINARY  WC_3_= BIOCHEMISTRY & MOLECULAR BIOLOGY  WC_4_=OR BIOLOGY  WC_5_=CHEMISTRY, MULTIDISCIPLINARY  WC_6_=ENGINEERING, CHEMICAL  WC_7_=GENETICS & HEREDITY  WC_8_=MATHEMATICS  WC_9_= MEDICINE, RESEARCH & EXPERIMENTAL  WC_10_=MICROBIOLOGY  WC_11_=NEUROSCIENCES  WC_12_=PARASITOLOGY  WC_13_= PHYSICS, MULTIDISCIPLINARY  WC_14_= PSYCHIATRY  WC_15_= PUBLIC, ENVIRONMENTAL & OCCUPATIONAL HEALTH  WC_16_= SOCIAL SCIENCES, INTERDISCIPLINARY  WC_17_= SOIL SCIENCE  WC_18_= TROPICAL MEDICINE  WC_19_= VETERINARY SCIENCES |
| **SO** “Publication name”  SO=(Acta Scientiae Veterinariae OR Animal Reproduction OR ARQUIVO BRASILEIRO DE MEDICINA VETERINARIA E ZOOTECNIA OR ARQUIVOS DE NEURO-PSIQUIATRIA OR ARQUIVOS DE NEURO-PSIQUIATRIA OR Bioscience Journal OR Bioscience Journal OR BRAGANTIA OR BRAZILIAN ARCHIVES OF BIOLOGY AND TECHNOLOGY OR BRAZILIAN JOURNAL OF BIOLOGY OR BRAZILIAN JOURNAL OF CHEMICAL ENGINEERING OR BRAZILIAN JOURNAL OF MEDICAL AND BIOLOGICAL RESEARCH OR BRAZILIAN JOURNAL OF MEDICAL AND BIOLOGICAL RESEARCH OR BRAZILIAN JOURNAL OF MICROBIOLOGY OR BRAZILIAN JOURNAL OF PHYSICS OR Brazilian Journal of Poultry Science OR BULLETIN OF THE BRAZILIAN MATHEMATICAL SOCIETY OR Cadernos de Saude Publica OR Cadernos de Saude Publica OR Ciencia & Saude Coletiva OR CIENCIA E AGROTECNOLOGIA OR DADOS-REVISTA DE CIENCIAS SOCIAIS OR GENETICS AND MOLECULAR BIOLOGY OR GENETICS AND MOLECULAR BIOLOGY OR GENETICS AND MOLECULAR RESEARCH OR GENETICS AND MOLECULAR RESEARCH OR JOURNAL OF THE BRAZILIAN CHEMICAL SOCIETY OR JOURNAL OF VENOMOUS ANIMALS AND TOXINS INCLUDING TROPICAL DISEASES OR Medicina Veterinaria-Recife OR MEMORIAS DO INSTITUTO OSWALDO CRUZ OR MEMORIAS DO INSTITUTO OSWALDO CRUZ OR Movimento OR PESQUISA AGROPECUARIA BRASILEIRA OR PESQUISA VETERINARIA BRASILEIRA OR QUIMICA NOVA OR REVISTA BRASILEIRA DE CIENCIA DO SOLO OR REVISTA BRASILEIRA DE PARASITOLOGIA VETERINARIA OR REVISTA BRASILEIRA DE PARASITOLOGIA VETERINARIA OR REVISTA BRASILEIRA DE PSIQUIATRIA OR REVISTA BRASILEIRA DE PSIQUIATRIA OR REVISTA BRASILEIRA DE ZOOTECNIA-BRAZILIAN JOURNAL OF ANIMAL SCIENCE OR REVISTA BRASILEIRA DE ZOOTECNIA-BRAZILIAN JOURNAL OF ANIMAL SCIENCE OR Revista Ciencia Agronomica OR Revista da Sociedade Brasileira de Medicina Tropical OR Revista de Psiquiatria Clinica OR REVISTA DE SAUDE PUBLICA OR REVISTA DE SAUDE PUBLICA OR REVISTA DO INSTITUTO DE MEDICINA TROPICAL DE SAO PAULO OR Saude e Sociedade OR SCIENTIA AGRICOLA OR Semina-Ciencias Agrarias) |
